# SARS-CoV-2 encoded ORF3a interacts with YY1 to promote latent HCMV reactivation

**DOI:** 10.1371/journal.ppat.1013344

**Published:** 2025-07-16

**Authors:** Sisi Xia, Xiaoping Huo, Nanfang Liu, Xinyu Liu, Tianyou Wang, Muyi Guo, Chi Zeng, Pin Wan, Jun Chen

**Affiliations:** 1 Department of Biological Engineering, Wuhan Polytechnic University, Wuhan, China; 2 State Key Laboratory of Bioactive Molecules and Druggability Assessment, Jinan University, Guangzhou, China; 3 Key Laboratory of Viral Pathogenesis & Infection Prevention and Control (Jinan University), Ministry of Education, Guangzhou, China; 4 Hubei Key Laboratory of Cognitive and Affective Disorders, Institute of Biomedical Sciences, School of Medicine, Jianghan University, Wuhan, China; State University of New York Upstate Medical University, UNITED STATES OF AMERICA

## Abstract

Human Cytomegalovirus (HCMV) is a commonly infected double-stranded DNA virus of the *β-herpesviridae* subfamily that typically establishes lifelong latency or persistent infection following primary infection. The regulation of HCMV latency and reactivation is governed by the chromatin structure at the viral major immediate early promoter (MIEP) within myeloid cells. Both cellular and viral factors play a role in regulating the reactivation of latent HCMV. Recently, it has been found that severe acute respiratory syndrome coronavirus 2 (SARS-CoV-2) promotes HCMV reactivation in the clinic; however, the mechanism remains unclear. In this study, we found that SARS-CoV-2 ORF3a can activate HCMV MIEP by interacting with Yin Yang 1 (YY1), an inhibitor of MIEP. This interaction leads to YY1 ubiquitin-dependent degradation and subsequently promotes the reactivation of latent HCMV, as well as the replication and proliferation of the virus. These findings reveal the molecular mechanism underlying the interaction between SARS-CoV-2 and HCMV during co-infection, providing a new theoretical basis for future prevention and treatment strategies against the co-infection of these two viruses.

## Author summary

Although the WHO has declared the COVID-19 pandemic over, the impact of the pandemic on people still exists, especially the long-term symptoms known as post-acute sequelae or long COVID found in some individuals. Studies have shown that co-infection with other viruses may be an important factor in the development of long COVID. In clinical practice, SARS-CoV-2 may also lead to the reactivation of latent HCMV, a prevalent herpesvirus. Meanwhile, reactivation from latency plays a crucial role in recurrent HCMV infection. However, previous research has primarily focused on understanding the mechanisms that establish viral latency. In this study, we discovered that the SARS-CoV-2 ORF3a protein activates the HCMV major immediate-early promoter (MIEP) by interacting with the MIEP inhibitor Yin Yang 1 (YY1). This interaction leads to ubiquitin-dependent proteasomal degradation of YY1, thus promoting the reactivation of latent HCMV. These findings provide the first direct laboratory evidence and elucidate the specific mechanism by which SARS-CoV-2 promotes HCMV reactivation from latency, highlighting ORF3a as a potential therapeutic target and providing new insights for therapeutic interventions for COVID-19 complications.

## Introduction

Human Cytomegalovirus (HCMV) is a double-stranded DNA virus of the *β-herpesviridae* subfamily that infects more than 90% of the population in developing countries [1]. HCMV primary infection is usually followed by lifelong latent or persistent infection [2]. In healthy individuals, the infection is often asymptomatic. Only when the immune function of infected individuals is suppressed will it be reactivated, leading to serious clinical manifestations, even fatal [3].

The latency-reactivation cycle is an important mechanism employed by herpesviruses to evade host immunity and ensure survival and propagation within the host [4,5]. The sites for HCMV latency *in vivo* are mainly myeloid cells such as CD14^+^ monocytes and their CD34^+^ progenitors, which participate in human circulation after differentiating into macrophages, dendritic cells (DCs), or peripheral blood monocytes, thereby accelerating the transmission of infection [6–8]. Once HCMV-infected monocytes differentiate into macrophages or DCs, the expression of viral immediate-early (IE) genes can be detected immediately [9]. This is then followed by viral replication and the release of infectious viral particles. Therefore, in myeloid cells, the activation of IE1 gene expression has become a marker of HCMV reactivation [10,11], and the chromatin structure of the major immediate-early promoter (MIEP), as the promoter of IE1, determines whether latency is maintained or reactivated [12,13].

The full length of MIEP is very complex and can be divided into five regions: the MIE promoter, the distal enhancer region, the proximal enhancer region, the unique region, and the modulator [14–16]. Within these regions, there are multiple binding sites for a diverse set of signal-regulated stimulatory or inhibitory transcription factors, such as NF-κB [17], ATF/CREB [18,19], activator protein 1 (AP-1) [20,21], Yin Yang 1 (YY1) [22], Sp1/Sp3 [23,24], the serum response factor (SRF) [25], Ets-2 repressor factor (ERF) [26], and growth factor independent 1 (Gfi-1) [27]. Therefore, the transcription of MIE is subject to complex regulation, and the promoter activity specific to cell type, cell division cycle, and differentiation state depends on the availability of appropriate transcription factors in MIEP regions [28,29].

Among these regulatory factors, the cellular transcription repressor YY1, a zinc finger protein that belongs to the GLI-Krüppel gene family, plays an important role in the latency of HCMV. During latent infection of undifferentiated cell lines, YY1 can bind to the negative regulatory elements of MIEP and inhibit its activity, and HCMV latency cannot be established in the absence of YY1 [22,30]. YY1 dimers generally occupy active enhancers and repress transcription by disrupting enhancer-promoter looping or through physical interactions with activator proteins [31,32]. The negative regulation of viral gene expression by YY1 has also been shown for other viruses [33,34]. It has been reported that the adenovirus E1A protein can bind to and alleviate the repression of YY1 on the adeno-associated virus P5 promoter [35]. However, there are currently limited reports on how HCMV relieves YY1-mediated inhibition of MIEP during the reactivation from latency to induce lytic infection.

Virome interactions are important determinants for viral disease [36,37]. The large-scale testing and screening during the COVID-19 pandemic have brought active research attention to the pathogenesis of co-infection [38]. Severe acute respiratory syndrome coronavirus type 2 (SARS-CoV-2), the novel β-coronavirus that caused the COVID-19 pandemic of the past few years, can cause severe human respiratory disease and even high mortality. Although COVID-19 is now self-limiting in most individuals, a proportion of those infected still experience long-term symptoms known as post-acute sequelae or long COVID [39]. It has been reported that pre-infection with influenza A virus (IAV) significantly enhances the infectivity of SARS-CoV-2 in various types of cells, possibly due to a unique characteristic of IAV that increases ACE2 expression [40]. The higher antibody responses to Epstein-Barr virus (EBV) lytic antigens measured in long COVID patients suggest that concurrent reactivation of EBV with SARS-CoV-2 infection could contribute to chronic disease [41]. Meanwhile, several clinical reports have emerged regarding the reactivation of HCMV in severe cases of COVID-19 [42–49]. The presence and reactivation of these chronic viral infections, such as HCMV, EBV, and HIV, are widely believed to be as potential contributors to long COVID and associated with distinct syndromic patterns [50]. However, the mechanism by which SARS-CoV-2 infection leads to the reactivation of these latently infected viruses, particularly HCMV, is rarely reported.

In this study, we cloned the full-length sequence of the HCMV immediate early gene promoter (MIEP) into a plasmid vector containing a luciferase reporter gene to construct a promoter-driven reporter gene plasmid. The SARS-CoV-2 ORF3a was then observed to effectively enhance the activation of MIEP, as indicated by screening with the dual-luciferase reporter assay. The ORF3a protein present in SARS-CoV-2 is located between the S and E proteins and is the largest accessory protein [51]. ORF3a plays a significant role in viral pathogenesis by facilitating viral assembly and release, as well as inducing cellular pro-inflammatory immune responses that can trigger COVID-19-related cytokine storms [52]. Further Co-immunoprecipitation (Co-IP) screening revealed that ORF3a can interact with the key cellular transcription repressor YY1 of HCMV MIEP and induce its ubiquitin-dependent degradation. Moreover, studies on post-mortem COVID-19 samples showed that viral RNA and antigens were detected in activated monocytes and macrophages [53,54]. Thus, we further validated the mechanism of co-infection reactivation in THP-1 model cells for HCMV latency. These findings highlight the first direct laboratory evidence and mechanistic explanation of co-infection between the two viruses, which will provide insights for the study and treatment of related sequelae caused by SARS-CoV-2 and HCMV-related disorders.

## Results

### Screening of SARS-CoV-2 protein that promotes MIEP activation

**Fig 1:**
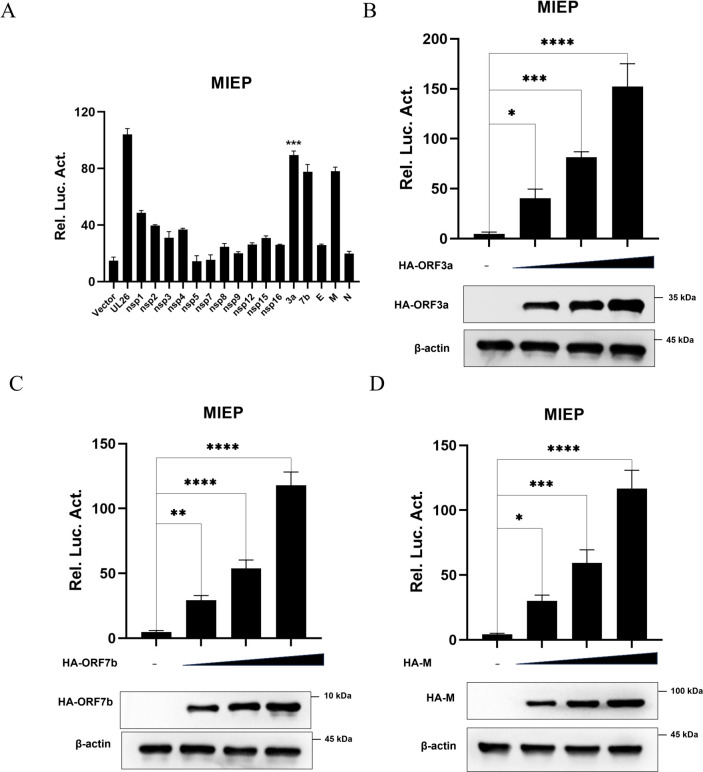
Screening of SARS-CoV-2 protein that promotes MIEP activation. **(A)** Dual luciferase assay was used to analyze the effect of SARS-CoV-2 viral proteins on MIEP in transfected cells. 293T cells were transfected with 500 ng pCAGGS-HA vector as empty control, or pCAGGS-HA-UL26 as positive control, or each pCAGGS-HA vector encoding various SARS-CoV-2 proteins (including NSP1, NSP2, NSP3, NSP4, NSP5, NSP6, NSP7, NSP8, NSP9, NSP12, NSP15, NSP16, ORF3a, ORF7b, E, M, and N), along with the pGL3-MIEP (500 ng) luciferase plasmid and pRL-TK (20 ng, as internal control). After 48 hours of transfection, the cells were harvested and analyzed using a dual-luciferase assay kit. (**B, C and D**) Dual luciferase assay was used to analyze the effect of ORF3a, ORF7b, M on MIEP in transfected cells. The pGL3-MIEP (500 ng), along with the control vector pRL-TK (20 ng), and each pCAGGS-HA vector encoding ORF3a, ORF7b, and M at increasing gradients (200/400/800 ng), were co-transfected into 293T cells, respectively. At 48 h after transfection, the cells were harvested and detected according to the dual luciferase detection kit. Data are presented as the means ± SD of three independent experiments. Differences were considered statistically significant when * indicates p < 0.05, ** indicates p < 0.01, *** indicates p < 0.001, and **** indicates p < 0.0001.

In order to explore the specific mechanism of SARS-CoV-2 on HCMV reactivation, we first investigated the effect of SARS-CoV-2-encoded proteins on HCMV MIEP activities. The firefly luciferase reporter gene plasmid containing HCMV MIEP (pGL3-MIEP), Renilla luciferase control reporter vector (pRL-TK), and the plasmid expressing the SARS-CoV-2 coding protein were co-transfected into 293T cells for 48 h, and the dual fluorescence reporter system was used to screen for SARS-CoV-2 proteins that may significantly affect the activity of MIEP. In addition, the plasmid expressing HCMV-encoded protein UL26, known to activate MIEP, was used as a positive control in this test [55]. The results showed that the relative fluorescence values of ORF3a, ORF7b, and M proteins of SARS-CoV-2 were significantly higher than those of the empty vector control. That is, ORF3a, ORF7b, and M proteins significantly enhanced the transcriptional activity of MIEP (Fig 1A).

In order to further determine the promoting effects of SARS-CoV-2 ORF3a, ORF7b and M proteins on HCMV MIEP initial transcription, 293T cells were transfected with pGL3-MIEP and co-transfected with gradient amounts of plasmid expressing ORF3a, ORF7b, and M proteins, respectively. Double luciferase reporter results showed that the promoting effects of ORF3a (Fig 1B), ORF7b (Fig 1C) and M (Fig 1D) proteins on the MIEP of HCMV enhanced with the increased concentration of transfection. Therefore, we preliminarily screened three SARS-CoV-2 coding genes that can activate the MIEP of HCMV, which may reactivate the latent HCMV infection during the co-infection of SARS-CoV-2 and HCMV.

### SARS-CoV-2 ORF3a interacts with YY1

**Fig 2:**
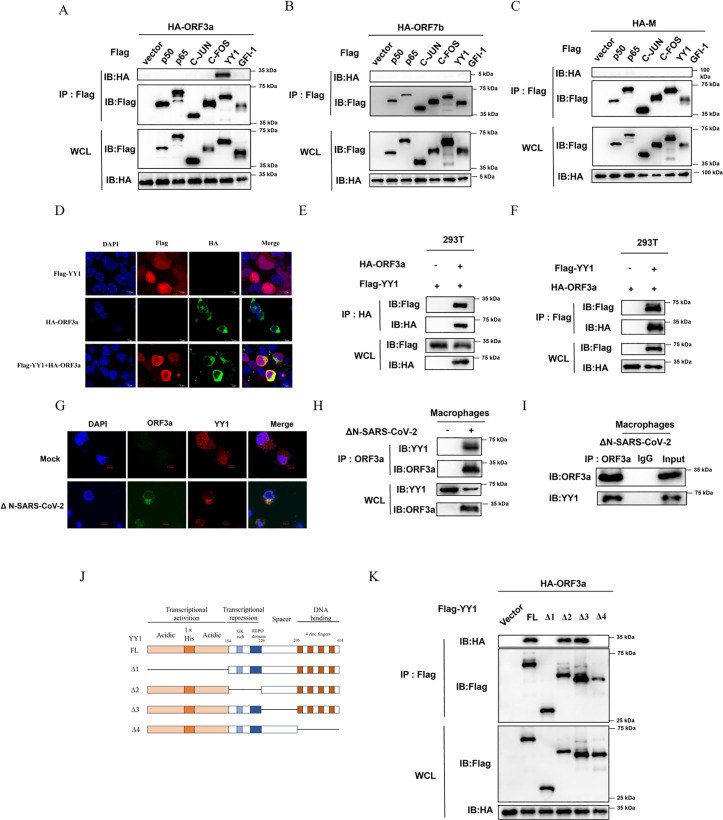
ORF3a of SARS-CoV-2 interacts with YY1. (**A, B and C**) Co-immunoprecipitation (Co-IP) analysis was conducted to investigate the association between ORF3a and the proteins p50, p65, c-jun, c-Fos, YY1, and Gfi-1 in transfected cells. ORF3a and the proteins p50, p65, c-jun, c-Fos, YY1, and Gfi-1 in transfected cells. pCAGGS-HA-ORF3a **(A)**, pCAGGS-HA-ORF7b (**B**) and pCAGGS-HA-M (**C**) and pcDNA3.1(+)-3 × Flag-p50, pcDNA3.1(+)-3 × Flag-p65, pcDNA3.1(+)-3 × Flag-c-jun, pcDNA3.1(+)-3 × Flag-c-Fos, pcDNA3.1(+)-3 × Flag-YY1, pcDNA3.1(+)-3 × Flag-Gfi-1 were co-transfected into 293T cells. At 48 h post-transfection, the supernatant of the cell lysate was collected for immunoprecipitation using anti-Flag antibodies. The resulting immunoprecipitants were subjected to heat denaturation and analyzed by Western blot with anti-HA or anti-Flag antibodies. **(D)** 293T cells were individually or collectively transfected with the pCAGGS-HA-ORF3a and the pcDNA3.1(+)-3 × Flag-YY1. At 48 h post-transfection, the cells were fixed and permeabilized, followed by incubation with anti-Flag and anti-HA primary antibodies, and Dylight 555/647-conjugated fluorescent secondary antibodies. The subcellular localization of ORF3a (green) and YY1 (red) was visualized, and cell nuclei stained using DAPI (blue). In the same field of view, the term “Merge” indicated the overlapping fluorescence from both the nuclei and the expressed proteins. **(E and F)** Co-immunoprecipitation (Co-IP) analysis was conducted to investigate the association between HA-ORF3a and YY1 in transfected cells. pCAGGS-HA-ORF3a and the pcDNA3.1(+)-3 × Flag-YY1 plasmids were co-transfected into 293T cells. At 48 h post-transfection, the supernatant of the cell lysate was collected for immunoprecipitation (IP) using anti-HA or anti-Flag antibodies. The immunoprecipitated samples were subsequently subjected to heat denaturation and analyzed by Western blot with anti-HA or anti-Flag antibodies. **(G)** THP-1 cells were infected with ΔN-SARS-CoV-2 (MOI = 5) for 48 h and then induced to differentiate into macrophages with TPA for 24 **h.** The cells were fixed and permeabilized, and then incubated with anti-YY1 and anti-ORF3a primary antibodies and Dylight 555/647 conjugated fluorescent secondary antibodies. The subcellular localization of ORF3a (green) and YY1 (red) was visualized, and nuclei were stained with DAPI (blue). In the same field of view, the term “Merge” indicates overlapping fluorescence of nuclei and expressed proteins. (**H and I**) Co-immunoprecipitation (Co-IP) was used to detect the interaction between endogenous YY1 and ORF3a in THP-1 cells. After THP-1 cells were infected with ΔN-SARS-CoV-2 (MOI = 5) for 48 h and then induced to differentiate into macrophages with TPA for 24 h, supernatants of cell lysates were immunoprecipitated with anti-ORF3a antibody. After heat denaturation of the co-immunoprecipitation complexes, Western blot analysis was performed using anti-YY1 and anti-ORF3a antibodies. **(J and K)** Co-immunoprecipitation (Co-IP) analysis was conducted to investigate the association between truncated clones of YY1 and ORF3a in transfected cells. pCAGGS-HA-ORF3a plasmids and the pcDNA3.1(+)-3 × Flag-YY1 truncated plasmid (pcDNA3.1(+)-3 × Flag-YY1-Δ1, Δ2, Δ3 or Δ4) were co-transfected into 293T cells. At 48 h post-transfection, the supernatant of the cell lysate was collected for immunoprecipitation (IP) using anti-Flag antibodies. The resulting immunoprecipitants were subjected to heat denaturation and subsequently analyzed by Western blot using anti-HA or anti-Flag antibodies. The displayed images were representative ones from three independent experiments.

Through the above screening, we preliminarily confirmed that the ORF3a, ORF7b, and M proteins of SARS-CoV-2 can significantly stimulate the MIEP of HCMV. It is known that MIEP is regulated by multiple factors, such as YY1, NF-κB and AP-1, which have been extensively studied [20,25,56–59]. To explore whether ORF3a, ORF7b, and M proteins regulate MIEP by interacting with these transcription factors, we successfully cloned some of the reported transcriptional regulators associated with MIEP and co-transfected them into 293T cells along with plasmids expressing ORF3, ORF7, or M encoded by SARS-CoV-2, respectively. After 48 hours of transfection, Co-immunoprecipitation (Co-IP) experiments were performed to screen and verify their interactions. Firstly, cell lysates were incubated with immobilized anti-Flag agarose beads to isolate the corresponding Flag-tagged protein complexes. Then, Western blot analyses using an anti-HA antibody were performed to identify the co-precipitated proteins. The results showed that co-expressed with Flag-YY1, HA-ORF3a efficiently immunoprecipitated Flag-YY1 in cells, indicating that ORF3a can form a complex with YY1 (Fig 2A). However, co-expression of ORF7b and M proteins with the aforementioned transcription factor proteins did not result in co-immunoprecipitation, indicating that the observed effects may be mediated through indirect mechanisms (Fig 2B and 2C).

To test whether the interaction between ORF3a and YY1 naturally occurs in the same cellular compartment or affects each other’s intracellular localization, we performed confocal assays of ORF3a and YY1 proteins in 293T cells. Flag-YY1 and HA-ORF3a were expression alone or co-expression into 293T cells through plasmid transfection. After 48 hours of transfection, they were detected by indirect immunofluorescence microscopy (IFA) using mouse anti-HA and rabbit anti-Flag antibodies. The results showed that YY1 was mainly concentrated in the nucleus and distributed relatively uniformly when transfected alone, while ORF3a was mainly present in the cytoplasm as previously reported [60]. However, co-expression increased the colocalization of the two proteins within the cytoplasm, which was further confirmed by our observation that ORF3a retained more YY1 in the cytoplasm through nuclear and cytoplasmic fractions separation experiments (Figs 2D and S1).

To further confirm the interaction between ORF3a and YY1, mutual Co-IP assays were performed in 293T cells. The plasmids expressing HA-ORF3a and Flag-YY1 were co-transfected into 293T cells. After 48 hours of transfection, the corresponding HA or Flag-tagged protein complexes were isolated by incubation of cell lysates with immobilized anti-HA or anti-Flag agarose beads. Subsequent Western blot analysis using an anti-Flag antibody showed that HA-ORF3a efficiently immunoprecipitated Flag-YY1 when co-expressed in cells and vice versa, thus further confirming the interaction between ORF3a and YY1. (Fig 2E and 2F). Next, the interaction between ORF3a and YY1 was further verified in THP-1-derived macrophages under SARS-CoV-2 infection. THP-1 cells were infected with ΔN-SARS-CoV-2 (ΔN recombinant SARS-CoV-2 virus like particles) for 48 hours and then induced to differentiate into macrophages by 12-o-tetradecanoyl phorbol 13-acetate (TPA) for 24 hours. Indirect immunofluorescence microscopy (IFA) was performed using anti-ORF3a and anti-YY1 antibodies. The results confirmed the colocalization of ORF3a and YY1 in the macrophage cytoplasm under viral infection (Fig 2G). In addition, Co-IP experiments were also performed in THP-1-derived macrophages using anti-ORF3a as the IP antibody to isolate the corresponding ORF3a protein complexes. The results showed that ORF3a is capable of precipitating endogenous YY1 from cell lysates with high efficiency, further confirming that ORF3a interacts with YY1 under physiological conditions (Fig 2H and 2I).

YY1 is composed of the following major domains: transcription activation domain (1–154 residues), transcription repression domain (155–226 residues), spacer domain (227–295 residues), and DNA binding domain-zinc finger (296–414 residues) [61]. To further map the regions of interaction between ORF3a and YY1, the plasmid expressing HA-ORF3a was transfected into 293T with pcDNA3.1-3 × Flag-Vector, pcDNA3.1-3 × Flag-YY1 FL (Full length) and YY1 deletion mutants Δ1, Δ2, Δ3 and Δ4 (Fig 2J), respectively. After 48 hours of transfection, the corresponding Flag-tagged protein complexes were isolated by adding immobilized anti-Flag agarose beads to the cell lysates. Subsequently, Western blot analysis using an anti-HA antibody showed that YY1Δ1 (deletion of the transcriptional activation domain) and YY1Δ4 (deletion of the zinc finger domain) lost the ability to interact with ORF3a (Fig 2K). These results indicate that the interaction between ORF3a and YY1 depends on both the transcriptional activation domain and the DNA-binding zinc finger domain of YY1.

### ORF3a restores the YY1-suppressed MIEP activity

**Fig 3:**
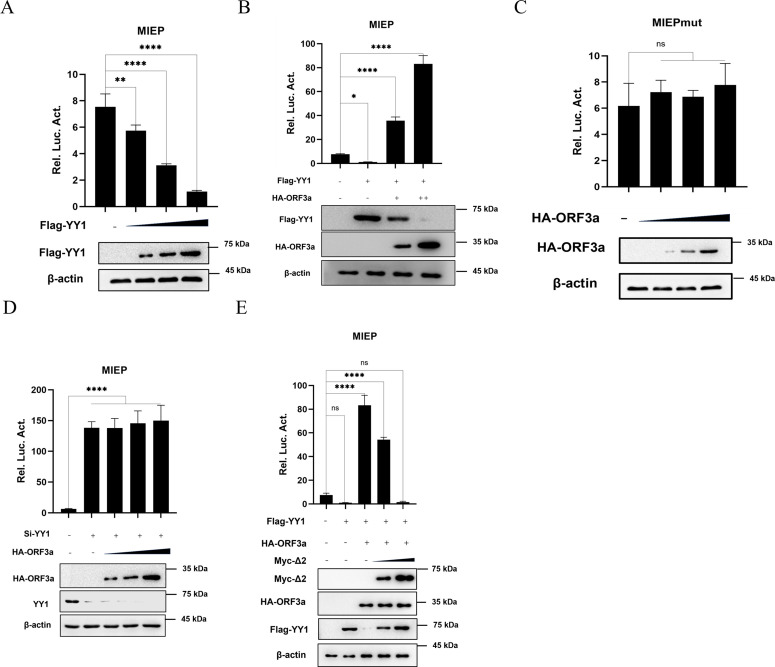
The inhibitory effect of YY1 on MIEP activity is restored by ORF3a in a dose-dependent manner. **(A)** Dual luciferase assay was used to analyze the effect of YY1 on MIEP in transfected cells. pGL3-MIEP (500 ng), the control vector pRL-TK (20 ng), and the gradient increasing pcDNA3.1(+)-3 × Flag-YY1 plasmid (200/400/800 ng) were co-transfected into 293T cells. At 48 h after transfection, the cells were harvested and detected according to the dual luciferase detection kit. **(B)** Dual luciferase assay was used to analyze the effect of ORF3a and YY1 on MIEP in the transfected cells. pGL3-MIEP (500 ng), control vector pRL-TK (20 ng), constant pcDNA3.1(+)-3 × Flag-YY1 plasmid and pCAGGS-HA-ORF3a plasmid in gradient increments (200/400/800 ng) were co-transfected into 293T cells. At 48 h after transfection, cells were collected and detected by dual luciferase detection kit. **(C)** The effect of ORF3a on MIEP with YY1 binding site mutation in transfected cells was analyzed by dual luciferase assay. The MIEP plasmid with YY1 binding site mutation (pGL3-MIEPmut) (500 ng), the control plasmid pRL-TK (20 ng), and pCAGGS-HA-ORF3a plasmids in gradient increments (200/400/800 ng) were transfected into 293T cells. After 48 hours of transfection, the cells were harvested and measured using a dual-luciferase assay kit. **(D)** The effect of ORF3a on MIEP in YY1 siRNA-knockdown cells was analyzed by dual-luciferase assay. After transfecting Si-YY1 into 293T cells for 24 hours, the pCAGGS-HA-ORF3a plasmid was transfected, and 48 hours later, the cells were harvested and measured using a dual-luciferase assay kit. **(E)** Dual luciferase assay was used to analyze the effect of ORF3a and YY1 on MIEP in the presence of YY1 Δ2 deletion mutant in the transfected cells. pGL3-MIEP (500 ng), control vector pRL-TK (20 ng), pcDNA3.1(+)-3 × Flag-YY1, pCAGGS-HA-ORF3a and pCMV-Myc-YY1-Δ2 plasmid (Myc-Δ2) in gradient increments (200/800 ng) were co-transfected into 293T cells. At 48 h after transfection, cells were collected and detected by dual luciferase detection kit. All images shown are representative of three independent experiments. Data are presented as the means ± SD of three independent experiments. Differences were considered statistically significant when * indicates p < 0.05, ** indicates p < 0.01, *** indicates p < 0.001, and **** indicates p < 0.0001.

To investigate whether the interaction between ORF3a and YY1 modulates MIEP activity, we first confirmed the inhibitory effect of YY1 on MIEP activity. pGL3-MIEP, the control vector pRL-TK, and gradient-increasing Flag-YY1 plasmids were co-transfection into 293T cells. At 48 hours after transfection, cells were collected and detected using dual luciferase detection kit. The results showed that MIEP activity gradually decreased with the increasing amount of YY1 transfection (Fig 3A).

To explore the role of ORF3a in this process, pGL3-MIEP, the control vector pRL-TK, a constant Flag-YY1 plasmid, and a gradient-increasing amounts of ORF3a plasmids were co-transfection into 293T cells. At 48 hours after transfection, the cells were harvested and detected according to the dual luciferase detection kit. We found that the inhibitory effect of YY1 on MIEP activity was gradually alleviated by increasing amounts of transfected ORF3a expression plasmid (Fig 3B). Meanwhile, it was noteworthy that an increase in the amount of HA-ORF3a expression vector appeared to lead to a reduction in YY1 levels, as observed in the immunoblot analysis. Moreover, we constructed pGL3-MIEPmut reporter plasmid that lacking the YY1 binding cis-elements in the MIEP according to previous report [23]. As shown in Fig 3C, results showed that ORF3a could no longer regulate MIEP when the YY1 binding site of MIEP was mutated. These results suggest that the activation of HCMV MIEP by ORF3a may depend on YY1 and its binding to the MIEP promoter.

To further confirm the role of interaction between ORF3a and YY1 in this process, the MIEP dual luciferase reporter assay was performed in 293T cells transfected with siRNA specifically targeting YY1. Our results showed that YY1 knockdown promoted MIEP activity; however, at the same time, the gradient-increase in HA-ORF3a expression vector transfection no longer promoted MIEP activity (Fig 3D). Next, in the co-transfection mixtures for the MIEP dual luciferase reporter assay, we added the YY1 Δ2 deletion mutation plasmid in a gradient manner. Before that, we confirmed that YY1 Δ2 mutant, which lacks the transcriptional repressor domain, could competitively bind to ORF3a, but had lost the ability to repress MIEP activity (S2 and S3 Figs). The results showed that the wild-type YY1 re-established its repression effect on MIEP when the YY1 Δ2 deletion mutant competed for ORF3a binding (Fig 3E). These findings further confirmed that the restoring of YY1-suppressed MIEP activity by ORF3a is dependent on its interaction with YY1.

### ORF3a promotes the Ubiquitin-dependent proteasomal degradation of YY1

**Fig 4:**
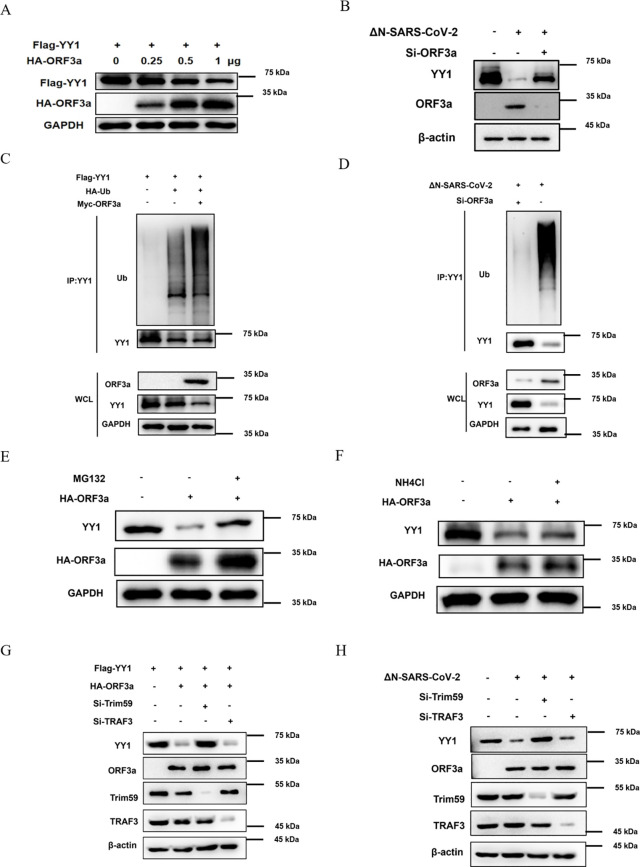
ORF3a of SARS-CoV-2 promotes the ubiquitination degradation of YY1. (**A**) pcDNA3.1(+)-3 × Flag-YY1 and gradient-increasing pCAGGS-HA-ORF3a plasmid were co-transfected into 293T cells. After 48 h, the cells were harvested and analyzed by Western blot using antibodies against the HA or Flag tags to detect the expression levels of YY1 and ORF3a. **(B)** After transfection of Si-ORF3a into THP-1 cells for 24 hours, the cells were infected with ΔN-SARS-CoV-2 (MOI = 5) for 48 h and then induced to become macrophages with TPA for 24 h. Cell lysates were thermally denatured and subsequently subjected to Western blot analysis using anti-YY1 or anti-ORF3a antibodies. (**C**) pcDNA3.1(+)-3 × Flag-YY1, pCMV-Myc-ORF3a, and pCAGGS-HA-Ub were co-transfected into 293T cells. After 48 h, the cells were then harvested and subjected to immunoprecipitation with a specific antibody against YY1. Western blot analysis was performed using antibodies against anti-Myc, anti-HA, or anti-Flag to evaluate YY1 expression and the effects of ubiquitination. **(D)** After transfection of Si-ORF3a into THP-1 cells for 24 hours, the cells were infected with ΔN-SARS-CoV-2 (MOI = 5) for 48 h and then induced to become macrophages with TPA for 24 **h.** The cells were collected for immunoprecipitation using specific antibody against YY1. Ubiquitination of endogenous YY1 was subsequently detected by immunoblotting using anti-Ub and β-actin antibodies. **(E and F)** 293T cells were transfected with the pCAGGS-HA-ORF3a plasmid and subsequently treated with MG132 or NH_4_Cl. Cells were harvested after 48 h, and the expression levels of endogenous YY1 were assessed by Western blot using either HA or YY1 antibodies. **(G)** 293T cells were treated with si-TRIM59 and si-TRAF3, and pCAGGS-HA-ORF3a and pcDNA3.1(+)-3 × Flag-YY1 plasmids were transfected into 293T cells. Cells were harvested after 48 h, and the expression level of YY1 in cells was determined by Western blot using HA or YY1 antibodies. **(H)** TRIM59 or TRAF3 siRNA-knockdown THP-1 cells and control cells were infected with SARS-CoV-2 (MOI = 5) for 48 h, and then were induced to differentiate into macrophages with TPA for 24 h. Cells were harvested and protein levels of endogenous YY1 were determined by immunoblotting using an anti-YY1 antibody. All images shown are representative of three independent experiments.

After confirming the interaction between SARS-CoV-2 ORF3a and YY1 in host cells, we further explored the effects of this interaction on YY1. As observed in 293T cells, the expression level of YY1 gradually decreased with the increasing gradient of ORF3a expression vector transfection (Fig 4A). Similarly, in SARS-CoV-2-infected macrophages, we observed that the protein expression of YY1 was significantly reduced in the presence of ORF3a; conversely, after ORF3a knockdown, the protein expression of YY1 returned to normal levels (Fig 4B).

These findings indicate that ORF3a has a certain degrading effect on YY1. To further investigate the mechanism of YY1 degradation mediated by ORF3a. Flag-YY1 and Myc-ORF3a plasmids were transfected into 293T cells with or without the addition of HA-Ub expression vector. Subsequently, for ubiquitination analysis, YY1 was first isolated by immunoprecipitation with anti-Flag antibody, and then the ubiquitinated YY1 was detected by Western blot using an anti-HA antibody. The results showed that the ubiquitination level of YY1 was significantly increased in the presence of ORF3a (Fig 4C). This result was further validated in SARS-CoV-2-infected macrophages. Macrophages were infected with ΔN-SARS-CoV-2 and then induced with TPA for 24 h. Cells were harvested and subjected to co-immunoprecipitation with YY1 specific antibody. The ubiquitination of endogenous YY1 was detected by immunoblotting using an anti-Ub antibody. We found that ORF3a significantly promoted the ubiquitination of endogenous YY1 (Fig 4D).

Next, to gain further insight into the ubiquitin degradation pathway. 293T cells were transfected with HA-ORF3a and treated with the proteasome inhibitor MG132 (Fig 4E) or the lysosome inhibitor NH_4_Cl (Fig 4F), respectively. The cells were collected and the expression of ORF3a and YY1 were detected by Western blot. The results showed that the promotion of YY1 degradation by ORF3a could be effectively inhibited by MG132, while the addition of NH_4_Cl had no effect. These results suggest that ORF3a promotes the ubiquitin-dependent degradation of YY1 through the proteasome pathway.

It has been reported that SARS-CoV-2 viral protein ORF3a injures renal tubules by interacting with an E3 ubiquitin ligase TRIM59 to induce STAT3 activation [62]. Not coincidentally, there is a report that the SARS-CoV ORF3a protein activates NF-κB and the NLRP3 inflammasome by interacting with another ubiquitin ligase TRAF3, and promoting TRAF3-dependent K63-linked ubiquitination of p105 and ASC [63]. To further explore which E3 ligases are involved in ORF3a-mediated YY1 degradation. 293T cells with TRIM59 or TRAF3 knockdown were transfected with HA-ORF3a and Flag-YY1 plasmids, respectively. After 48 hours of transfection, cells were collected and the expression of ORF3a and YY1 was detected by Western blot. The results demonstrated that ORF3 was unable to induce YY1 degradation when TRIM59, but not TRAF3, was specifically knocked down using target-specific siRNA (Fig 4G). Similarly, in SARS-CoV-2-infected macrophages, we observed a significant reduction in the protein expression of YY1 in the presence of ORF3a; however, knockdown of TRIM59, but not TRAF3, restored the protein expression of YY1 to the normal level (Fig 4G). These results suggest that ORF3a mediates the ubiquitination degradation of YY1 through TRIM59.

### SARS-CoV-2 ORF3a promotes the reactivation of HCMV from latency

**Fig 5:**
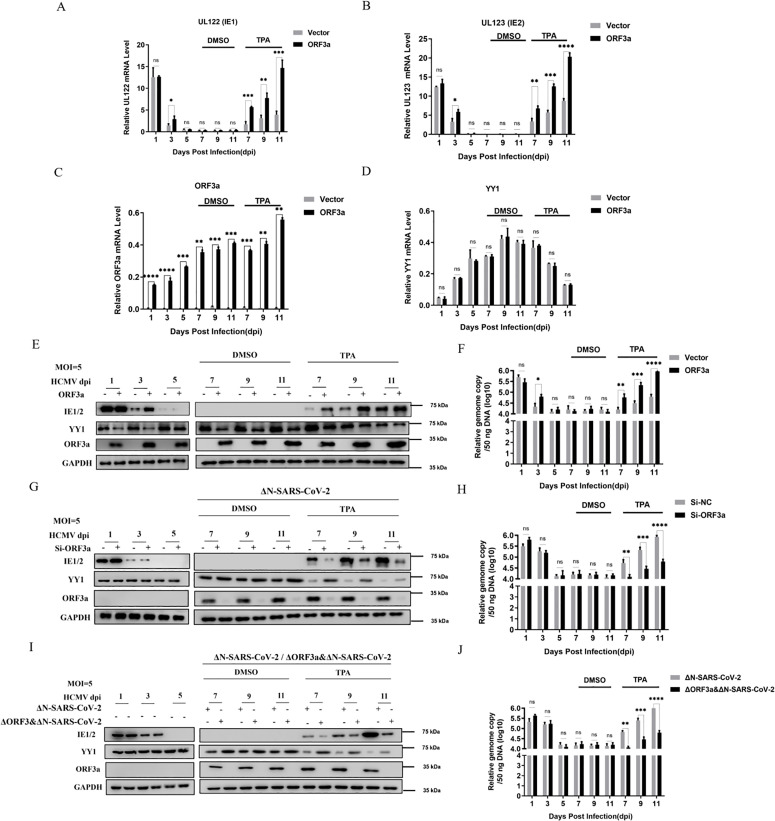
ORF3a of SARS-CoV-2 promotes HCMV reactivation from latency. (**A, B, C, D, E, and F**) THP-1 cells stably expressing pLenti-3 × Flag-vector and pLenti-3 × Flag-ORF3a were infected with the HCMV-Towne strain (MOI = 5) for 5 days. Then, TPA was added at a final concentration of 100 ng/mL to induce the differentiation of THP-1 monocytes into macrophages. Cell samples were collected at 1, 3, 5, 7, 9, and 11 days of culture, with DMSO serving as a control. The mRNA expression levels of UL122 (**5A**), UL123 (**5B**), ORF3a (**5C**), and YY1 (**5D**) were measured by RT-qPCR. The expression of IE1/2, ORF3a, and YY1 proteins was detected by Western blot (**5E**), and the viral genome copy number was detected (**5F**). **(G and H)** After transfection of Si-ORF3a into THP-1 cells for 24 hours, the cells were infected with the HCMV-Towne strain at a multiplicity of infection of 5 for 5 days. They were then infected with ΔN-SARS-CoV-2 (MOI = 5). Then, TPA at a final concentration of 100 ng/mL was added to induce the differentiation of THP-1 monocytes into macrophages. Cell samples were collected at 1-, 3-, 5-, 7-, 9-, and 11-days post-treatment with DMSO as the control. The expression of IE1/2, ORF3a, and YY1 proteins was detected by Western blot (**5G**), and the viral genome copy number was detected (**5H**). **(I and J)** THP-1 cells and negative control cells were infected with the HCMV-Towne strain at a multiplicity of infection of 5 for 5 days. They were then infected with ΔN-SARS-CoV-2 or ΔORF3a & ΔN-SARS-CoV-2 (MOI = 5). Then, TPA at a final concentration of 100 ng/mL was added to induce the differentiation of THP-1 monocytes into macrophages. Cell samples were collected as control group at 1, 3, 5, 7, 9 and 11 days after treatment. The expression of IE1/2, ORF3a and YY1 protein was detected by Western blot (**5I**), and the viral genome copy number (**5J**) was detected. All images shown are representative of three independent experiments. Data are presented as the means ± SD of three independent experiments. Differences were considered statistically significant when * indicates p < 0.05, ** indicates p < 0.01, *** indicates p < 0.001, and **** indicates p < 0.0001.

Taken together, these results demonstrated that ORF3a promotes the ubiquitination and degradation of YY1, a key transcriptional repressor of MIEP. MIEP is an important switch for HCMV latency and reactivation; therefore, we further validated the role of ORF3a in the reactivation of HCMV from latency and in the context of ΔN-SARS-CoV-2 co-infection. THP-1 cells were used as a model system to establish HCMV latency and to stimulate reactivation. Firstly, THP-1 cells stably transfected with pLenti-3 × Flag-vector and were infected with the HCMV-Towne strain for 5 days. Then, TPA at a final concentration of 100 ng/mL was added to induce the differentiation of THP-1 monocytes into macrophages. Cell samples were collected at 1, 3, 5, 7, 9, and 11 days of culturing, and DMSO was used as the control. The mRNA levels of UL122 (IE1) and UL123 (IE2), as the main characteristic genes of HCMV MIEP transcription, were detected by RT-qPCR (Fig 5A and 5B). The results showed that IE1/2 mRNA expression gradually decreased during the first 5 days of infection, and finally reached and maintained a very low level of detection, indicating that the virus had entered the latent period. Interestingly, although ORF3a did not impact the final latent state, we observed that IE1/2 mRNA decreased at a slower rate in THP-1 cells stably transfected with ORF3a expression vector compared to the empty vector control groups. Moreover, IE1/2 mRNA expression was significantly increased in ORF3a stably transfected THP-1 cells during TPA-induced HCMV reactivation from latency. Meanwhile, we also verified the mRNA expression levels of ORF3a (Fig 5C) and examined the expression of YY1 (Fig 5D). As can be seen, YY1 mRNA expression was significantly increased during virus latency and decreased during virus reactivation, which is consistent with previous reports [64]. Meanwhile, stable transfection of ORF3a did not affect YY1 transcription. Next, we detected the protein levels of IE1/2, ORF3a, and YY1 proteins by Western blot (Fig 5E). The trends of IE1/2 protein levels were consistent with the RT-qPCR results described above; however, stable transfection of ORF3a expression vector significantly reduced the protein levels of YY1, which is consistent with the mechanism we explored above. In addition, the viral genome copy number during the reactivation from latency was also examined. Similarly, the results showed that ectopically expressed ORF3a obviously promoted the replication progress of HCMV during latent HCMV reactivation (Fig 5F). These results suggest that the ORF3a of SARS-CoV-2 promotes HCMV reactivation by down-regulating of YY1.

The effect of ORF3a on latent HCMV reactivation was further investigated under co-infection conditions. THP-1 cells with ORF3a knockdown and negative control cells were infected with the HCMV-Towne strain at a multiplicity of infection of 5 for 5 days. They were then infected with ΔN-SARS-CoV-2. At the same time, TPA at a final concentration of 100 100 ng/mL was added to induce the differentiation of THP-1 monocytes into macrophages. Cell samples were collected at 1, 3, 5, 7, 9, and 11 days of culturing with DMSO as the control. The expression levels of IE1/2, ORF3a and YY1 proteins were detected by Western blot. Although several studies have shown that monocytes and monocyte-derived macrophages do not express ACE2 and are primarily infected *in vitro* through an ACE2-dependent pathway, resulting in many viral genes expression being blocked and abortive replication of SARS-CoV-2 [65,66], we found that ORF3a could be efficiently expressed in THP-1 cells and THP-1-induced macrophages infected with ΔN-SARS-CoV-2 virus like particles. Moreover, it can be seen that in the context of co-infection, the protein levels of YY1 were increased when ORF3a was knocked down (Fig 5G). Furthermore, we also conducted a further detection of the viral genome copy number. The results were consistent with those confirmed above, and ORF3a expressed by the virus significantly promoted the replication progress of HCMV after the reactivation from latency (Fig 5H). To further verify the role of ORF3a under physiological conditions, the THP-1-derived macrophages as HCMV latency and reactivation model, were infected with ΔORF3a &ΔN-SARS-CoV-2 (ΔORF3a and ΔN recombinant SARS-CoV-2 virus like particles). The results showed that upon deletion mutation of ORF3a, the enhancing effect of SARS-CoV-2 on IE1/2 was markedly reduced (Fig 5I). The measurement of viral gene copy number further confirmed that the promotion of HCMV replication by ΔORF3a-SARS-CoV-2 was significantly attenuated during reactivation from latency (Fig 5J). These results further indicate that SARS-CoV-2 ORF3a triggers the degradation of YY1, which in turn enhances the expression of IE1/2, and ultimately facilitates the reactivation of latent HCMV.

## Discussion

Severe acute respiratory syndrome coronavirus 2 (SARS-CoV-2) is a highly infectious and pathogenic virus that can cause severe respiratory diseases in humans. Clinically, it has been found that co-infection of SARS-CoV-2 with various of pathogens can cause more severe symptoms, such as co-infection with hepatitis B virus (HBV), which causes liver dysfunction with bile duct cell damage [67], and co-infection with influenza virus, which causes more severe symptoms [68]. Of note, the reactivation of *Cytomegalovirus* and other herpesvirus families ranges from 20 to 70% in critically ill patients with COVID-19 and is associated with an increased risk of secondary infections and death. Existing research data show that HCMV blood reactivation occurs frequently in critically ill patients with COVID-19, leading to severe clinical manifestations and even death. Although the above clinical data suggest the existence of co-infection of SARS-CoV-2 and HCMV, the mechanism of action has rarely been reported. Here, we constructed a full-length promoter luciferase reporter plasmid that contains five regulatory regions of MIEP. Then, through dual-luciferase reporter assays, we screened SARS-CoV-2 open reading frame 3a (ORF3a) and found it efficiently enhances MIEP activation. Further Co-IP screening showed that ORF3a could specifically interact with the key cellular transcription repressor Yin Yang 1 (YY1), a critical inhibitor of HCMV MIEP. It has been reported that the fusion and invasion of THP-1 cell-derived macrophages by SARS-CoV-2 predominantly occur via an ACE2-independent pathway mediated by CD169, a type I lectin specific to myeloid cells [69]. Therefore, we further explored the mechanism of this interaction and the role of ORF3a in latent HCMV reactivation in THP-1 model cells co-infected with SARS-CoV-2. In this study, we provided the first direct laboratory evidence and mechanistic explanation that SARS-CoV-2 ORF3a activates HCMV MIEP by interacting with YY1, which leads to the ubiquitin-dependent degradation of YY1, and in turn promotes the reactivation of HCMV from latency. Of note, we also found that the ORF7b and M proteins could promote MIEP activation, as screened by dual-luciferase reporter assays. However, Co-IP experiments showed that ORF7b and M proteins did not interact with MIEP-related transcription factors, suggesting that their effects may be indirect. It has been reported that ORF7b functions in suppressing IFN-interferon production during early infection to facilitate evasion of host detection and induction of cell apoptosis by promoting the expression of TNF-α and IL-6 [70–72]. M protein inhibits the production of type I and type III interferons by targeting RIG-I/MDA-5 signaling and can be sensed by NLRP12 to promote an inflammatory response [73,74]. Considering that previous studies have shown that immunosuppressive and inflammatory signals may contribute to MIEP activation [20,57,75], and since the MIEP region contains multiple NF-κB recognition sites, we speculate that whether the activation of MIEP by ORF7b and M proteins through these two aspects needs to be further confirmed by follow-up studies. Even so, it remains to be further explored whether they play a role in HCMV latency and reactivation.

ORF3a of SARS-CoV-2 is a multifunctional protein that plays significant roles in viral replication and pathogenesis [51,52,76]. It is well established that ORF3a promotes viral assembly and release [77]. Notably, recent studies have also shown that ORF3a is associated with host inflammation and immunity, and may be one of the important factors contributing to the COVID-19-related cytokine storm [52]. It was reported that ORF3a triggers the secretion of proinflammatory factors by activating the NLRP3 inflammasome, which in turn leads to hyperinflammatory responses [78,79]. *In vitro* cellular studies have shown that ORF3a can independently induce NF-κB-mediated activation of proinflammatory cytokines (TNFα and IL-6) [80]. In animal models, loss of ORF3a reduced the risk of cytokine storm [81]. However, the specific mechanism of ORF3a in causing the COVID-19 related cytokine storm is still unclear and needs further study. Here, we identified a novel ORF3a-interacting protein YY1, which is an important transcriptional repressor in host cells. YY1 has been reported not only as a driver of many cancers [82–84], but also as a transcription factor regulating systemic inflammation [85–87]. In view of the possible antagonistic relationship between ORF3a and YY1 in inflammation and cellular immunity, their interaction may play an important regulatory role in the occurrence of cytokine storm caused by SARS-CoV-2 infection, which will be further explored in future studies.

YY1 is an important cellular transcriptional repressor and therefore a potential target for regulating many physiological processes. It has been reported that SMAD4/6 is activated by BMPR2 to inhibit TGF-β receptor signaling and induce YY1 degradation via the cellular microRNA hsa-miR-29a, which leads to the failure of HCMV to maintain latency [64]. In addition, Krippner Heidenreich *et al*. found two cleavage sites in the full-length YY1 sequence that are recognized by caspase [88]. In response to various apoptotic stimuli during apoptosis, the cleavage at these two sites leads to its rapid translocation into the nucleus, thus participating in the positive feedback loop of apoptosis. In this study, we demonstrated for the first time that the SARS-CoV-2-encoded protein ORF3a can interact with YY1, leading to its ubiquitination and degradation and playing an important role in the reactivation from latency of HCMV. Intriguingly, it has been reported that SARS-CoV-2 viral protein ORF3a injures renal tubules by interacting with an E3 ubiquitin ligase TRIM59 to induce STAT3 activation [62]. Not coincidentally, there is a report that the SARS-CoV ORF3a protein activates NF-κB and the NLRP3 inflammasome by interacting with another ubiquitin ligase, TRAF3, and promoting TRAF3-dependent K63-linked ubiquitination of p105 and ASC [63]. Here, our results show that YY1 is a novel target for ORF3a degradation and that TRIM59 is the specific E3 ubiquitin ligase responsible for ORF3a-induced YY1 ubiquitination, through which it regulates YY1 function during the reactivation from HCMV latency.

Previous studies have demonstrated that YY1 is highly expressed in the latent phase of HCMV infection and acts as a crucial factor for HCMV latency [64]. Our study further supports these findings. Moreover, we observed that YY1 expression diminishes during HCMV reactivation, suggesting that YY1 plays an important regulatory role in the process of HCMV reactivation from latency. However, it is worth reflecting that although SARS-CoV-2 co-infection promotes HCMV reactivation in the clinic, our results suggest that SARS-CoV-2 infection alone is not sufficient to cause HCMV reactivation in THP-1 model cells, and TPA is still required to induce cell differentiation, leading to HCMV reactivation. We speculate that SARS-CoV-2 co-infection leads to the reactivation from latency of HCMV not only through alteration of a single transcription factor of MIEP, but its reactivation may also require the joint influence of many factors *in vivo*. In addition, there are always some limitations in laboratory conditions; therefore, we need better physiological models to verify our findings, as well as a more comprehensive exploration of the mechanism by which SARS-CoV-2 co-infection leads to HCMV reactivation in clinical settings. While the THP-1 monocyte-derived macrophage differentiation system has been widely validated as a robust *in vitro* model for studying the mechanisms of HCMV latency-reactivation [10,89]. It would be interesting to conduct the experiments in primary cell types known to support latent HCMV infection. Indeed, CD34^+^ hematopoietic progenitor cells have been used as primary latently infected cells in some studies [90]. However, the scarcity of primary cells (especially CD34^+^ hematopoietic progenitor cells) and the technical difficulty of obtaining them for many laboratories have caused great limitations in practical experimental verification. Studying HCMV infection of CD34^+^ HPCs *in vitro* is also challenging, given the difficulty of maintaining progenitors in an undifferentiated state in culture. Goodrum *et al.* have developed an *in vitro* model to study HCMV latency in primitive CD34^+^ HPC populations. However, primary CD34^+^ HPCs must be infected immediately after isolation and especially cultured in long-term bone marrow culture on the immortalized murine AFT024 stromal cell layer, which is also unusual under physiological conditions [91].

Besides, following the pandemic, there has generally been an increased concern about the long-term effects of COVID-19 caused by SARS-CoV-2 infection [92–94]. HCMV is a herpesvirus that is widely latent in the human population. Thus, it is worth further exploration and research to consider if the reactivation from latency of HCMV due to SARS-CoV-2 co-infection could be an important characteristic and cause of long-COVID-19 complications. In conclusion, this study elucidated the interaction between SARS-CoV-2 and HCMV co-infection and highlighted ORF3a as a potential therapeutic target, providing new insights for therapeutic intervention of COVID-19 complications.

## Materials and methods

### Cells and viruses

Human foreskin fibroblasts (HFFs) and human embryonic kidney 293T cells (293T) were purchased from the American Type Culture Collection (Manassas, VA, USA). HFFs and 293T cells were cultured in Dulbecco’s Modified Eagle’s Medium (DMEM) supplemented with 10% fetal bovine serum (FBS) and 1% penicillin/streptomycin whereas THP-1 cells were cultured in RPMI 1640, supplemented as indicated above in a 5% CO_2_ incubator at 37°C. THP-1 cells were induced by 100 ng/mL TPA for 24 hours to become macrophages. Human cytomegalovirus strain Towne was provided by Professor Fenyong Liu from the University of California, Berkeley, USA. HCMV Towne was propagated in HFFs, and viral titers were quantified using a plaque-forming assay as previously described [95].

### The system of SARS-CoV-2 virus-like particles

The full-length SARS-CoV-2 GFP/ΔN cDNA and Caco-2 cells stably expressing the SARS-CoV-2 N gene derived from viral genome (MN908947 strain) were provided by Professor Ding Qiang, Tsinghua University, Beijing, China [96]. Then, the viral RNA transcript transcribed *in vitro* was electroporation into SARS-Cov-2 N gene stably expressed Caco-2 cells. Within 48 h, GFP fluorescence was observed, and after 96 h, cytopathic effect (CPEs) was observed, indicating the production and reproduction of recombinant SARS-CoV-2 virus like particles (designed as ΔN-SARS-CoV-2). Similarly, the full-length SARS-CoV-2 ΔORF3a & ΔN cDNA was constructed based on the full-length SARS-CoV-2 GFP/ΔN cDNA using overlap PCR technology. The produced recombinant SARS-CoV-2 virus like particles (designed as ΔORF3a & ΔN-SARS-CoV-2). Cell culture supernatants were obtained and centrifuged at 1000 g for 10 min to remove cell fragments. Then, using 0.22 μM filter membrane filter them. The viruses were aliquoted into tubes and stored at -80°C [97].

### Plasmids design and construction

The ORF3a gene was amplified from the cDNA genome of SARS-CoV-2 GFP/ΔN (MN908947) and cloned into the pCAGGS expression vector with HA tag at N terminus. The plasmids encoding the SARS-CoV-2 proteins were in the pCAGGS-HA vector containing the full-length coding region of each SARS-CoV-2 protein. pcDNA3.1(+)-3 × Flag-YY1 plasmid was constructed by PCR amplification of the YY1 ORF from reverse-transcribed cDNA of THP-1 cells and then cloned into the pcDNA3.1(+) expression vector with a 3 × Flag tag at the N terminus. pcDNA3.1(+)-3 × Flag-YY1-Δ1, Δ2, Δ3, and Δ4 were provided by Professor Bixiang Zhang from the Huazhong University of Science and Technology. The sequence of the plasmid previously constructed and stored in the laboratory was confirmed by sequencing.

The full length of the major IE enhancer promoter containing nucleotides -1150 to +50 relative to the transcription initiation site was amplified by PCR from the bacterial artificial chromosome (BAC)-based clone of HCMV Towne strain and then inserted into XhoI and HindIII sites of pGL3-Basic vector (Promega) (designated as pGL3-MIEP). The primers used for PCR were:

pGL3-MIEP-F: ACGCTCGAGCTATATGCTGCAGTGAATAATA
pGL3-MIEP-R: ACGAAGCTTCGGAGGCTGGATCGGTCCCGG

The MIEP reporter plasmid lacking the YY1 binding site [22] was designated as pGL3-MIEPmut. This plasmid was constructed using the mutation primers and the Fast Site-Directed Mutagenesis kit (Transgen Biotech, Beijing, China; Cat. No. FM111–01) according to the manufacturer’s protocol. The mutation primers are as follows:

F: TTACATAACTTACGGCAAC GCCCGCCTGGCTGACCGCC
R: CGGTCAGCCAGGCGGGCCGGTTGCGTAAGT

Table 1 lists the constructs generated in this study.

**Table 1:** Plasmid constructs used in the study.

Plasmids	Description	Reference /source
pcDNA3.1(+)-3 × Flag-YY1	pcDNA3.1(+)-3 × Flag containing YY1 full-length coding region	This study
pCMV-Myc-ORF3a	pCMV-Myc containing SARS-CoV-2 ORF3a full-length coding region	This study
pCAGGS-HA-ORF3a	pCAGGS-HA containing SARS-CoV-2 ORF3a full-length coding region	This study
pcDNA3.1(+)-3 × Flag-c-jun	pcDNA3.1(+)-3 × Flag containing c-jun full-length coding region	This study
pcDNA3.1(+)-3 × Flag-c-Fos	pcDNA3.1(+)-3 × Flag containing c-Fos full-length coding region	This study
pcDNA3.1(+)-3 × Flag-Gfi-1	pcDNA3.1(+)-3 × Flag containing Gfi-1 full-length coding region	This study
pcDNA3.1(+)-3 × Flag-p50	pcDNA3.1(+)-3 × Flag containing p50 full-length coding region	This study
pcDNA3.1(+)-3 × Flag-p65	pcDNA3.1(+)-3 × Flag containing p65 full-length coding region	This study
pCAGGS-HA-UL26	pCAGGS-HA containing HCMV UL26 full-length coding region	This study
pGL3-MIEP	pGL3 containing HCMV MIEP sequence	This study
pCAGGS-HA-Ub	pCAGGS-HA containing Ub full-length coding region	This study
pCMV-Myc-Δ2	pCMV-Myc containing YY1-Δ2 coding region	This study
pLenti-3 × Flag-ORF3a	pLenti-3 × Flag containing SARS-CoV-2 ORF3a full-length coding region	This study
pGL3-MIEPmut	pGL3 containing HCMV MIEP sequence which lacking the YY1 binding site	This study

### Luciferase reporter assay

The pGL3-MIEP luciferase reporter plasmid was transfected into 293T cells for 48 hours using Lipofectamine 2000 Reagent (Life Technologies; Cat. No. 11668019). Luciferase activity was analyzed using a dual luciferase reporter system (Promega; Cat. No. E1960). Data were normalized to Renilla luciferase activity.

### Reagents and antibodies

The antibodies used for Western blot were as follows: GAPDH antibody (Yeasen, Shanghai, China; Cat. No. 30201ES60; diluted 1:10,000), DYKDDDDK antibody (Flag; Proteintech, Wuhan, China; Cat. No. 66008–3-Ig; diluted 1:3,000), HA antibody (Proteintech, Wuhan, China; Cat. No. 51064–2-AP; diluted 1:3,000), ORF3a antibody (ABclonal, Wuhan, China; Cat. No. A20234; diluted 1:3,000), YY1 antibody (Proteintech, Wuhan, China; Cat. No. 66281–1-Ig; diluted 1:5,000), Ub antibody (Proteintech, Wuhan, China; Cat. No. 10201–2-AP; diluted 1:1,000), Histone H3 antibody (Proteintech, Wuhan, China; Cat. No. 17168–1-AP; diluted 1:3,000), β-actin antibody (Proteintech, Wuhan, China; Cat. No. 66009–1-Ig; diluted 1:10,000), Myc antibody (Proteintech, Wuhan, China; Cat. No. 60003–2- Ig; clone 1A5A2; diluted 1:3,000), IgG (Proteintech, Wuhan, China; Cat. No. B900620). IE1/IE2 antibody (Abcam, Cambridge, England; Cat. No. ab53495; diluted 1:1,000),

The inhibitors used are as follows: MG132 (MedChemExpress, Monmouth Junction, NJ, USA; Cat. No. HY-13259) was used at a concentration of 10 µM, and NH_4_Cl at a concentration of 20 mM (MedChemExpress, Monmouth Junction, NJ, USA; Cat. No. HY-1269C). Phorbol 12-myristate 13-acetate (TPA) (MedChemExpress, Monmouth Junction, NJ, USA; Cat. No. HY-18739) was used at a concentration of 100 ng/mL.

### siRNA design and transfection

The siRNA oligos for gene silencing were purchased from Sangon Biotech (Shanghai, China). Transient transfection of siRNAs was performed using the Lipofectamine 3000 reagent according to the manufacturer’s instructions. The following siRNAs were used in this study:

control siRNA, 5’-TGGTTTACATGTCGACTAA-3’;
ORF3a siRNA, 5’-GAGAATCTTCACAATTGGAACTGTA-3’ [98];
control siRNA, 5’- CACGATAAGACAATGTATTT-3’;
TRAF3 shRNA, 5’- GGAAGAUUCGCGACUACAAGC-3’ [99];
Si-YY1, 5’-GCACAAAGATGTTCAGGGA-3’ [100];
TRIM59 shRNA, 5’-GGAAGCTGTTCTCCAGTAT-3’ [101].

Plasmid transfection was undertaken using Lipo293 transfection reagent (Beyotime, Shanghai, China; Cat. No. C0521) according to the manufacturer’s protocol.

### RNA isolation and RT-qPCR analysis

Total RNA was extracted from cell pellets using the Total RNA kit (Yeasen, Shanghai, China; Cat. No. 19221ES50). cDNA was prepared using HiScript II 1st Strand cDNA Synthesis Kit (Vazyme, Nanjing, China; Cat. No. R223-01). RT-qPCR was employed to detect mRNA expression levels on a CFX96 Real-Time System (Bio-Rad, Hercules, CA, USA), utilizing the SYBR Green Master Mix Kit (Yeasen, Shanghai, China; Cat. No. 11201ES08). The RT-qPCR cycling program was as follows: an initial denaturation stage of 95°C for 3 min; an amplification stage of 95°C for 20 s, 60°C for 30 s, and 72°C for 30 s for 40 cycles; and a final extension stage of 72°C for 5 min. A melting-curve analysis was conducted after each run to verify the specificity of the amplification reaction. GAPDH was used as the standard for data, and the relative expression of the target gene level was calculated as 2^-ΔΔCt^.The primers used in this study were as follows:

GAPDH-F: AAGGCTGTGGGCAAGG
GAPDH-R: TGGAGGAGTGGGTGTCG;
UL122 (IE1)-F: TTATTAAACCGCCCGTGCCT
UL122 (IE1)-R: TTCACCCTGTTCTTCCTCGC;
UL123 (IE2)-F: CCAAGAGAAAGATGGACCCTG
UL123 (IE2)-R: AACATAGTCTGCAGGAACGTC;
YY1-F: GAGGGATACCTGGCATTG
YY1-R: TTCTTGGAGCATCATCTTCT;
ORF3a-F: TGTTGGCGTTGCACTTCTTG
ORF3a-R: AGCAACGAGCAAAAGGTGTG.

### Immunofluorescence assay

The cells in the confocal dishes were gently washed with ice-cold PBS and then fixed using 4% paraformaldehyde for 20 minutes at room temperature. Next, the cells underwent three washes with ice-cold PBS (5 minutes each) before being permeabilized with 1 × PBS-T containing 0.2% Triton X-100 for 10 minutes. Following this, the cells were washed again with PBS and blocked with 1 × PBS-B containing 0.4% BSA for 30 minutes at 37°C. The cells were then incubated with the specified primary antibodies overnight at 4°C. Afterward, they were incubated with Alexa Fluor 555 and Alexa Fluor 647 conjugated secondary antibodies (diluted 1:1000) for one hour. DAPI was applied for nuclear staining (5 minutes), and the samples were sealed with 50% glycerin. Finally, images were captured using a Leica SP8 TCP confocal microscope. Quantitative analyses of the immunofluorescence images were conducted using a 63 × objective and LAS X software, based on three representative images.

### Western blot analysis

Cells were lysed in Triton X-100 lysis buffer containing a protease inhibitor cocktail (1:100 dilution) for 30 minutes on ice, and subsequently centrifuged at 16,000 × g for 10 minutes at 4°C. The supernatants were then mixed with 6 × SDS loading buffer, boiled for 10 minutes, and stored at -80°C. The samples were separated using SDS-PAGE and transferred onto polyvinylidene difluoride (PVDF) membranes. The membranes were blocked with 5% nonfat milk in 1 × TBST for 1 hour at room temperature with agitation. Following this, the membranes were incubated with the specified primary antibodies, followed by incubation with HRP-conjugated secondary antibodies.

### qPCR detection of HCMV DNA levels

The DNeasy tissue kit (Qiagen, Hilden, Germany) was adopted to extract total DNA from mock or HCMV-infected cells. Quantification of intracellular viral DNA levels was accomplished by qPCR amplification of the HCMV UL83 DNA region, using a pair of primers plus a specific TaqMan probe [102]. Three times of repetitions were carried out for each set of assays in triplicate. The shown data are Means ± SD from one representative experiment.

### Co-immunoprecipitation (Co-IP)

The cells were transfected with pCAGGS-HA-ORF3a or pcDNA3.1(+)-3× Flag-YY1. After 48 hours of transfection, the cells were washed and lysed on ice for 30 minutes using Triton X-100 lysis buffer (20 mM Tris-HCl, pH 7.5; 100 mM NaCl; 0.5 mM EDTA; 5% glycerol; 1% Triton X-100) supplemented with a 1 × protease inhibitor cocktail. Following vortexing at 4°C for 1 hour, the lysed cells were centrifuged to obtain the supernatants, which were then incubated with the indicated antibodies (anti-HA and anti-Flag from Sigma) and protein A/G affinity beads at 4°C for 4 hours with gentle shaking. Subsequently, the beads were centrifuged, washed three times with ice-cold PBS, boiled in 2 × SDS loading buffer for 10 minutes, and analyzed by Western blot using the indicated antibodies (anti-HA and anti-Flag).

### Nuclear and cytoplasmic separation experiments

Nuclear and cytoplasmic protein extraction kits (Beyotime, Shanghai, China; Cat. No. P0028) were used for nuclear and cytoplasmic components extraction as described below: Cells were washed twice with precooled 1 × PBS, then removed by scraping with a cell scraper and placed in EP tubes, centrifuged at 1000 rpm for 5 min at 4°C, and the precipitate was retained at the end of centrifugation. 100 µl cytoplasmic protein extraction reagent A (Cytoplasmic protein extraction reagent A: PMSF = 100:1) was added to the precipitate, and the precipitate was completely suspended by vortex vigorously for 5 s. The EP tube was then placed on ice for 15 min, followed by 10 µl of cytoplasmic protein extraction reagent B (Cytoplasmic protein extraction reagent B: PMSF = 100:1), followed by vortex for 5 s and an ice bath for 1 min. Then, after centrifugation at 16000 g for 5 min at 4°C, the supernatant was used as cytoplasmic protein. The precipitate was added to 50 µl of nucleoprotein extraction reagent and vortexed for 30 minutes. The cells were then centrifuged at 16000 g for 10 min at 4°C, and the supernatant at this time was nuclear protein.

### Statistical analyses

All data are shown as the mean and standard deviation of at least three independent experiments (details are provided in the table and figure legends). Statistical significance of the differences between groups was estimated using two-tailed Student’s t-test or one-way ANOVA using GraphPad Prism 6. P values were depicted as follows: ns, no significance; *, P < 0.05; **, P < 0.01; ***, P < 0.001.

## Supporting information

S1 Fig Localization of ORF3a and YY1 analyzed by nucleoplasm separation assay. 293T cells were transfected with pCAGGS-HA-ORF3a, pCAGGS-HA-ORF3a, or a combination of pCAGGS-HA-ORF3a and pcDNA3.1(+)-3 × Flag-YY1 plasmids. After 48 hours, the cell lysates were collected and the cytoplasmic and nuclear fractions were prepared using a nuclear and cytoplasmic protein extraction kit. Separated samples were subsequently heat-denatured and analyzed by Western blot. All images shown are representative of three independent experiments.
(TIF)

S2 Fig Effect of YY1 deletion mutants Δ2 on MIEP. Dual luciferase assay was used to analyze the effect of YY1 deletion mutant (pCMV-Myc-YY1-Δ2) on MIEP in transfected cells. The pGL3-MIEP plasmid, the control vector pRL-TK, and the YY1 deletion mutant (pCMV-Myc-YY1-Δ2) at increasing gradients were transfected into 293T cells. After 48 hours of transfection, the cells were collected and detected using a dual-luciferase assay kit. All images shown are representative of three independent experiments. Differences were considered statistically significant when * indicates p < 0.05, ** indicates p < 0.01, and *** indicates p < 0.001. **** indicates p < 0.0001.
(TIF)

S3 Fig YY1 deletion mutants Δ2 competitively bind ORF3a. Co-immunoprecipitation (Co-IP) was used to analyze the correlation between HA-ORF3a, YY1 and YY1 deletion mutant (pCMV-Myc-YY1-Δ2) in transfected cells. pCAGGS-HA-ORF3a and pcDNA3.1(+)-3 × Flag-YY1 were co-transfected into 293T cells with gradient increasing YY1 deletion mutant (pCMV-Myc-YY1-Δ2). At 48 h after transfection, the supernatant of cell lysate was collected and immunoprecipitation (IP) was performed using anti-HA antibody. Immunoprecipitated samples were subsequently heat-denatured and analyzed by Western blot with anti-HA, anti-Myc and anti-Flag antibodies. All images shown are representative of three independent experiments.
(TIF)

S4 Fig Full western blot analyses. (PDF)

S1 Data Source data files. (XLSX)
