# Supplementary figures and images for: SARS-CoV-2 encoded ORF3a interacts with YY1 to promote latent HCMV reactivation
Source: PLoS Pathog. 2025 Jul 16;21(7):e1013344. doi: 10.1371/journal.ppat.1013344 (PMC12324675; doi:10.1371/journal.ppat.1013344)

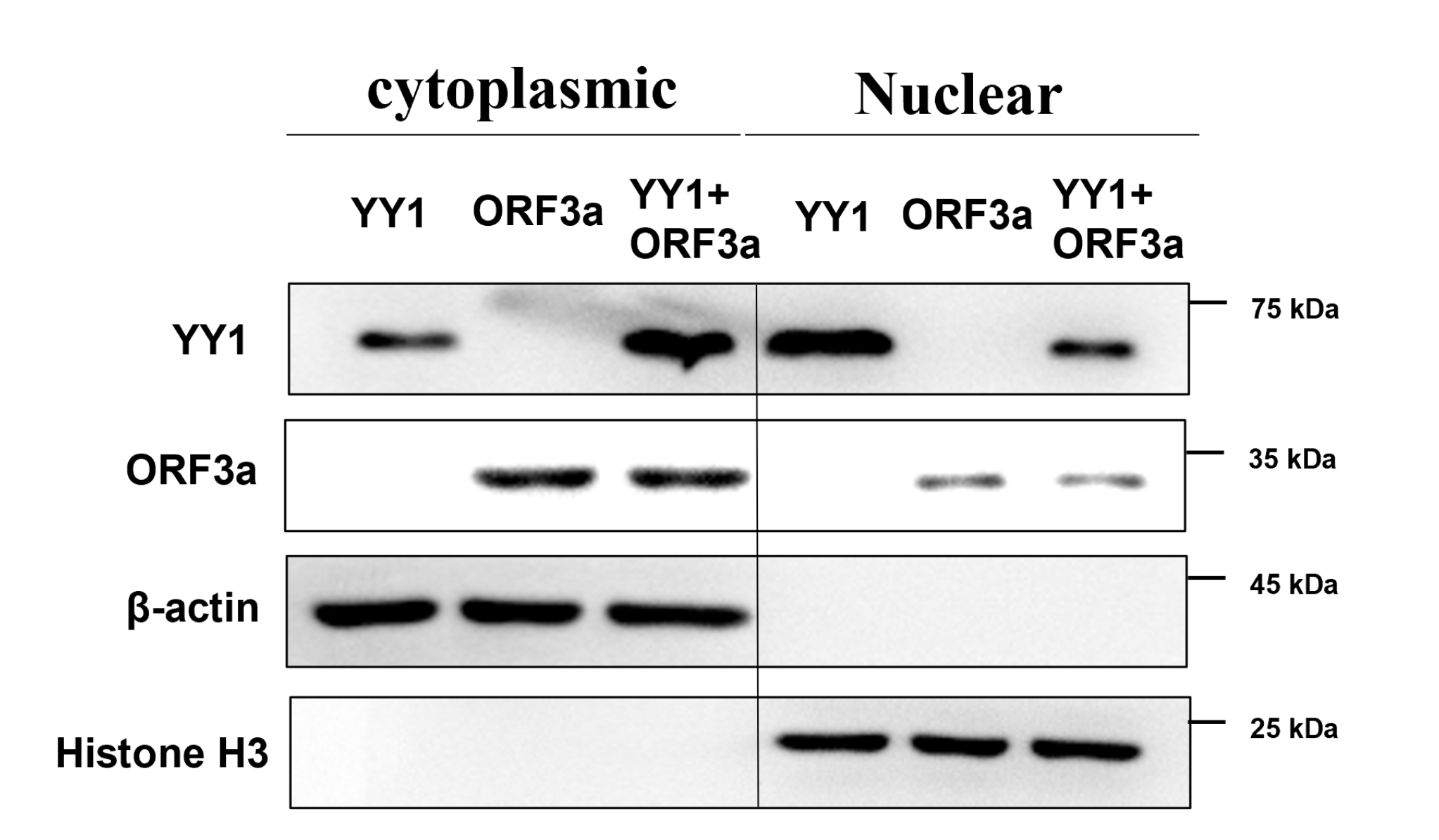

Supplement: S1 Fig — 293T cells were transfected with pCAGGS-HA-ORF3a, pCAGGS-HA-ORF3a, or a combination of pCAGGS-HA-ORF3a and pcDNA3.1(+)-3 × Flag-YY1 plasmids. After 48 hours, the cell lysates were collected and the cytoplasmic and nuclear fractions were prepared using a nuclear and cytoplasmic protein extraction kit. Separated samples were subsequently heat-denatured and analyzed by Western blot. All images shown are representative of three independent experiments. (TIF) [file ppat.1013344.s001.tif]

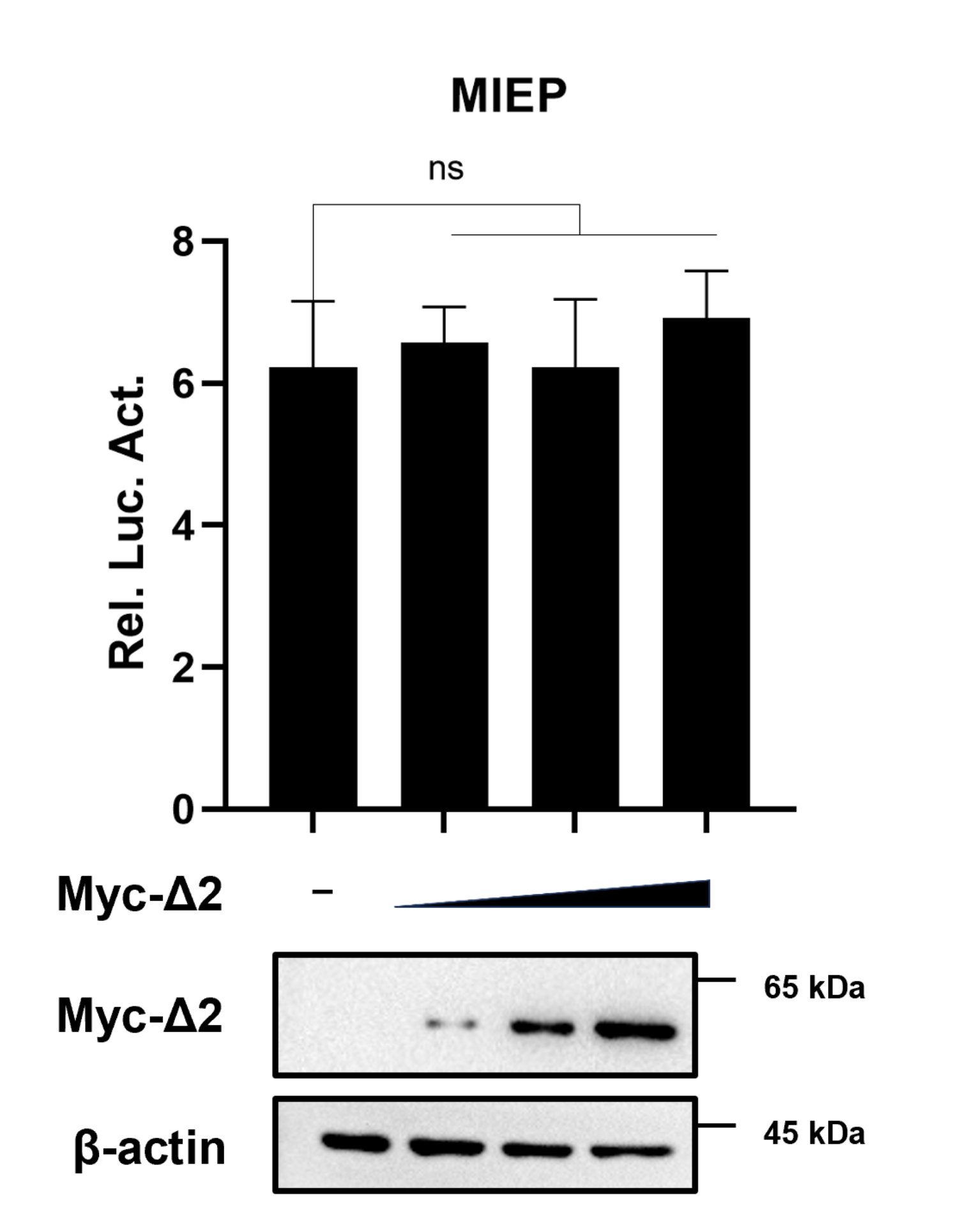

Supplement: S2 Fig — Effect of YY1 deletion mutants Δ2 on MIEP. Dual luciferase assay was used to analyze the effect of YY1 deletion mutant (pCMV-Myc-YY1-Δ2) on MIEP in transfected cells. The pGL3-MIEP plasmid, the control vector pRL-TK, and the YY1 deletion mutant (pCMV-Myc-YY1-Δ2) at increasing gradients were transfected into 293T cells. After 48 hours of transfection, the cells were collected and detected using a dual-luciferase assay kit. All images shown are representative of three independent experiments. Differences were considered statistically significant when * indicates p < 0.05, ** indicates p < 0.01, and *** indicates p < 0.001. **** indicates p < 0.0001. (TIF) [file ppat.1013344.s002.tif]

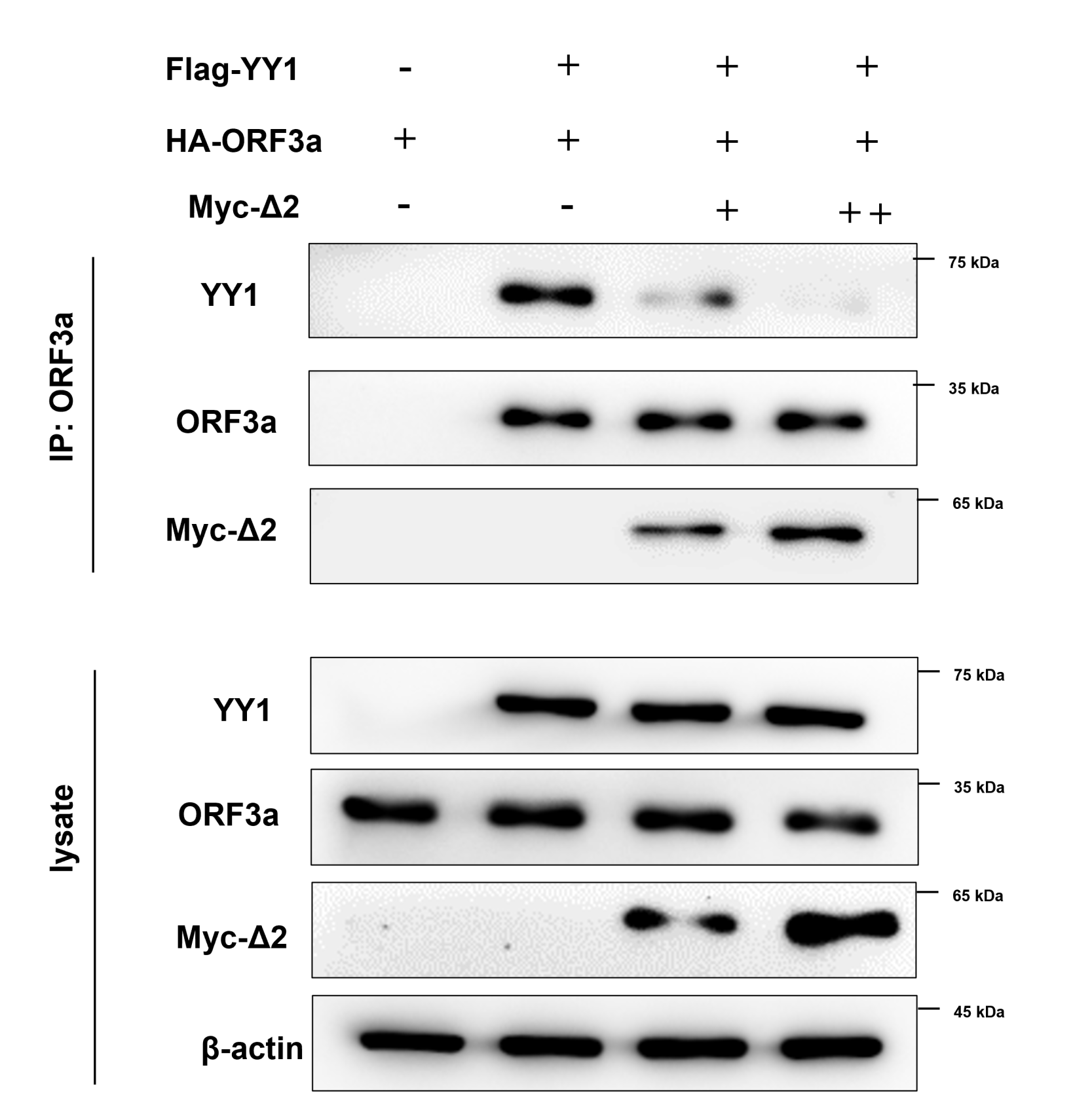

Supplement: S3 Fig — YY1 deletion mutants Δ2 competitively bind ORF3a. Co-immunoprecipitation (Co-IP) was used to analyze the correlation between HA-ORF3a, YY1 and YY1 deletion mutant (pCMV-Myc-YY1-Δ2) in transfected cells. pCAGGS-HA-ORF3a and pcDNA3.1(+)-3 × Flag-YY1 were co-transfected into 293T cells with gradient increasing YY1 deletion mutant (pCMV-Myc-YY1-Δ2). At 48 h after transfection, the supernatant of cell lysate was collected and immunoprecipitation (IP) was performed using anti-HA antibody. Immunoprecipitated samples were subsequently heat-denatured and analyzed by Western blot with anti-HA, anti-Myc and anti-Flag antibodies. All images shown are representative of three independent experiments. (TIF) [file ppat.1013344.s003.tif]
