# Supplementary material for: SARS-CoV-2 encoded ORF3a interacts with YY1 to promote latent HCMV reactivation
Source: PLoS Pathog. 2025 Jul 16;21(7):e1013344. doi: 10.1371/journal.ppat.1013344 (PMC12324675; doi:10.1371/journal.ppat.1013344)
Supplement: S4 Fig — (PDF) [file ppat.1013344.s004.pdf]

Figure B in Figure 1

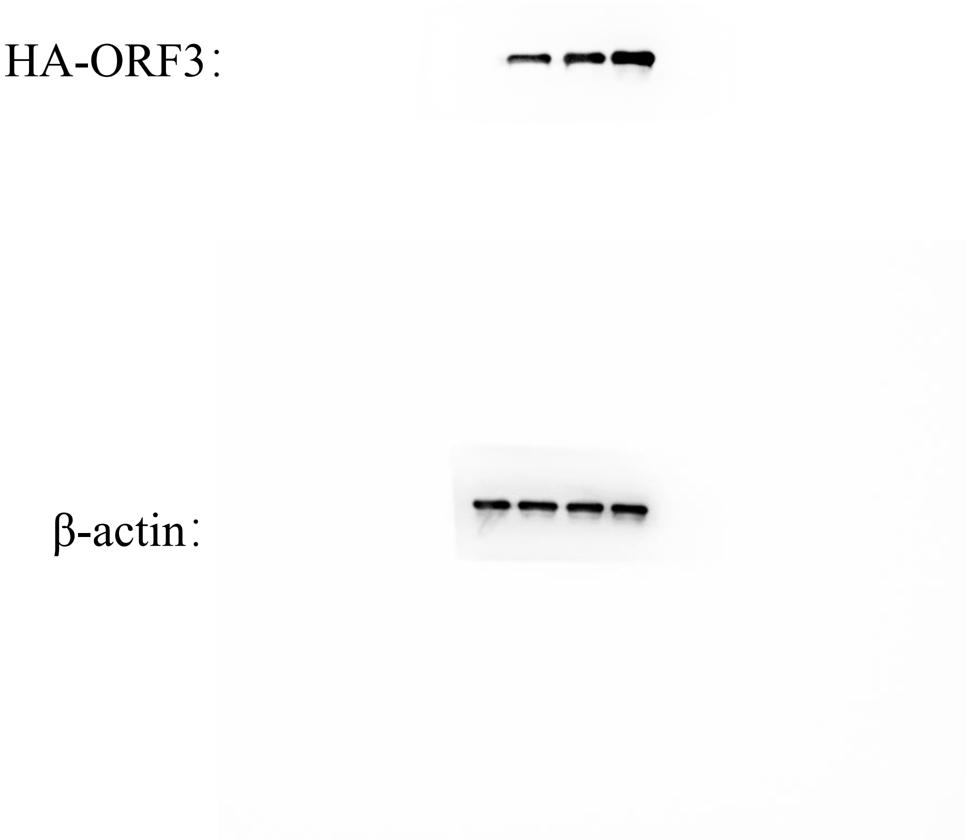

B

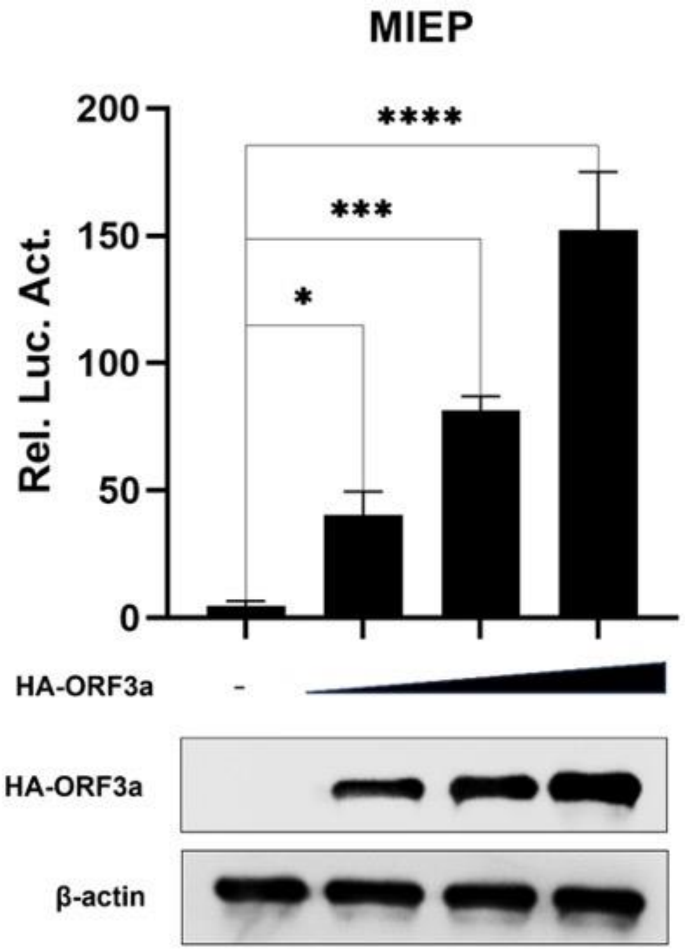

Figure C in Figure 1

HA-ORF7b：

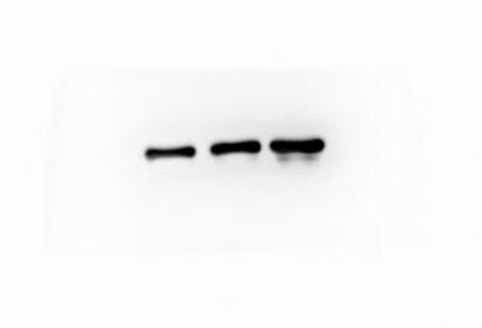

β-actin：

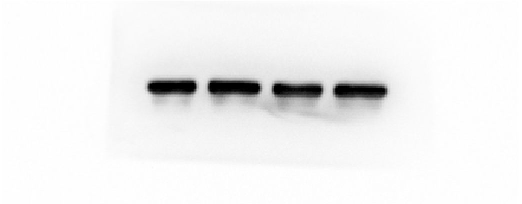

C

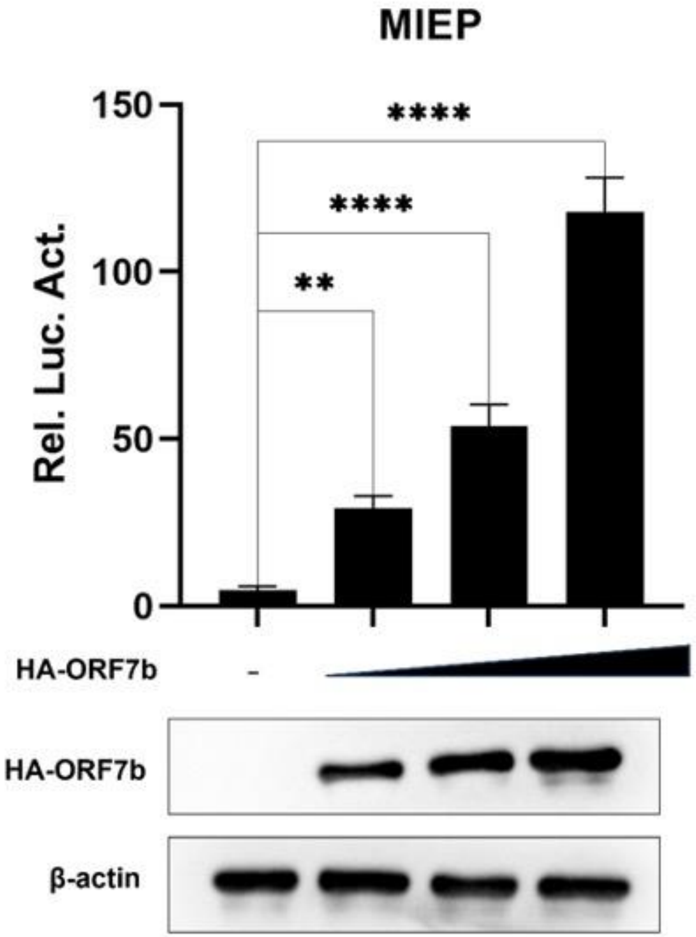

Figure D in Figure 1

HA-M：

β-actin：

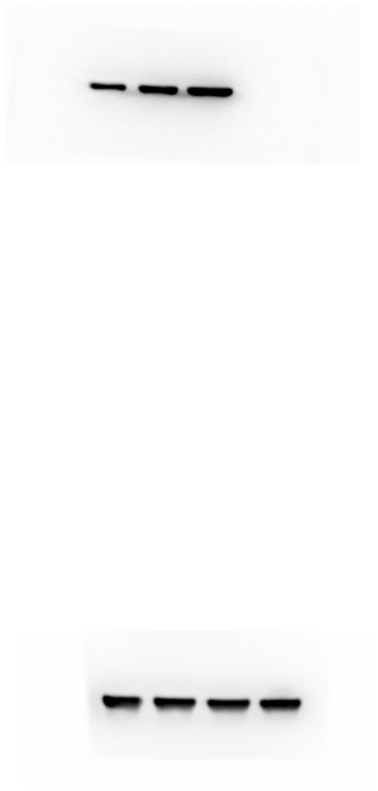

D

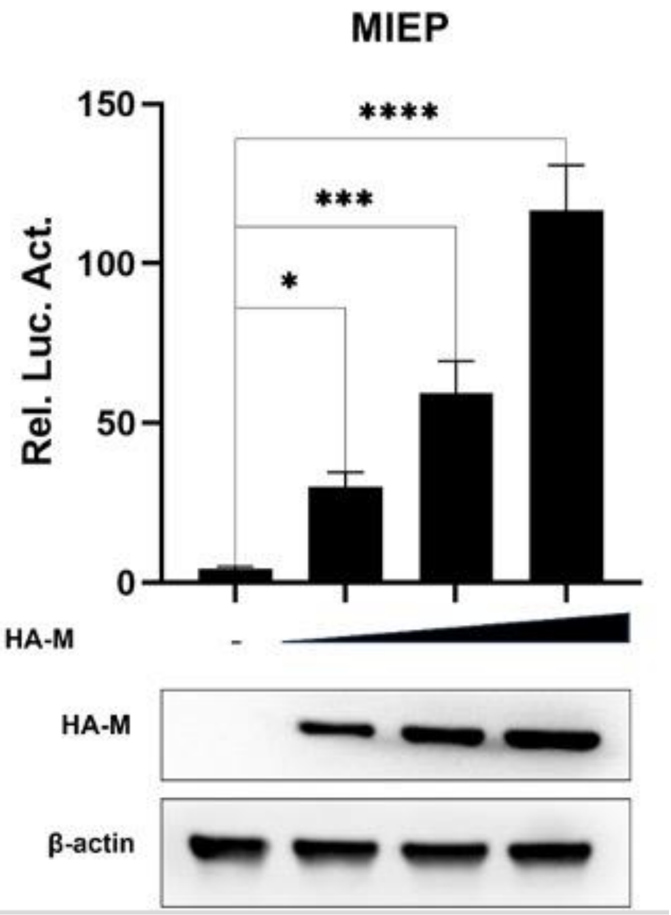

Figure A in Figure 2

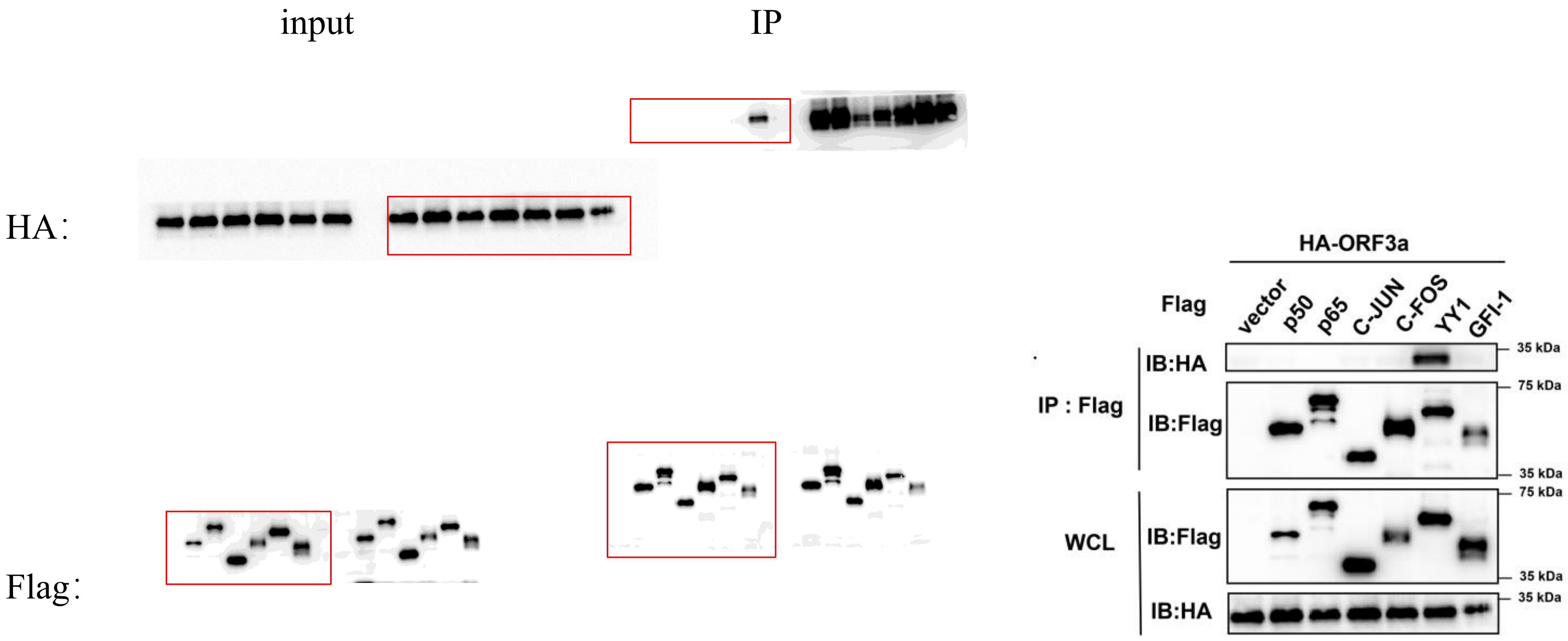

Figure B in Figure 2

input

IP

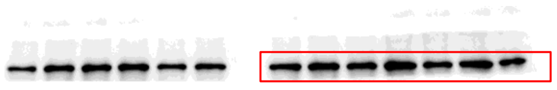

HA：

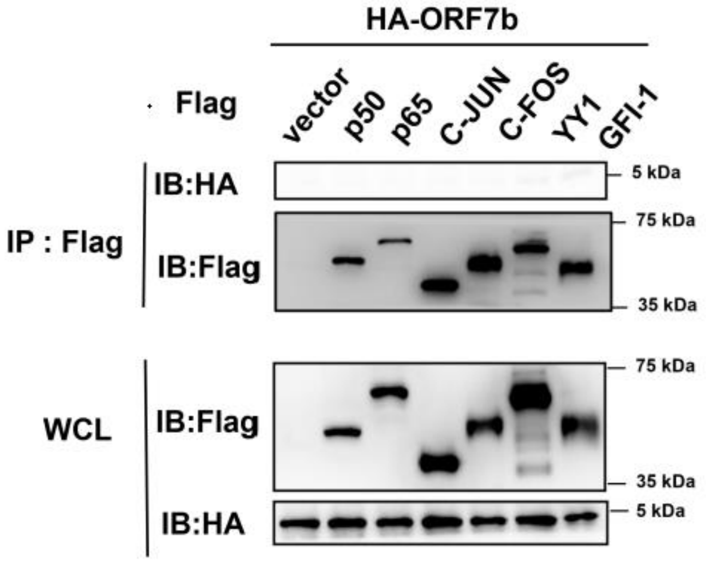

Flag：

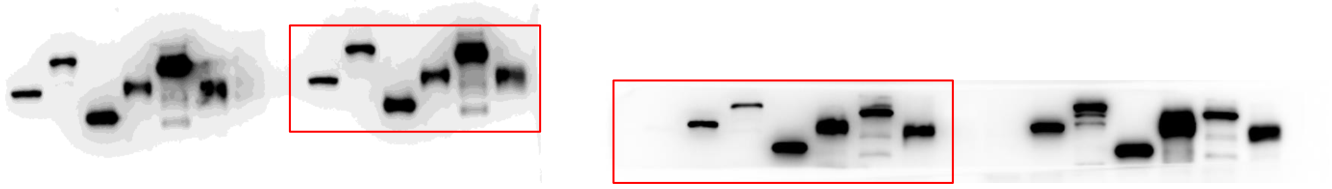

Figure C in Figure 2

input

IP

HA：

Flag：

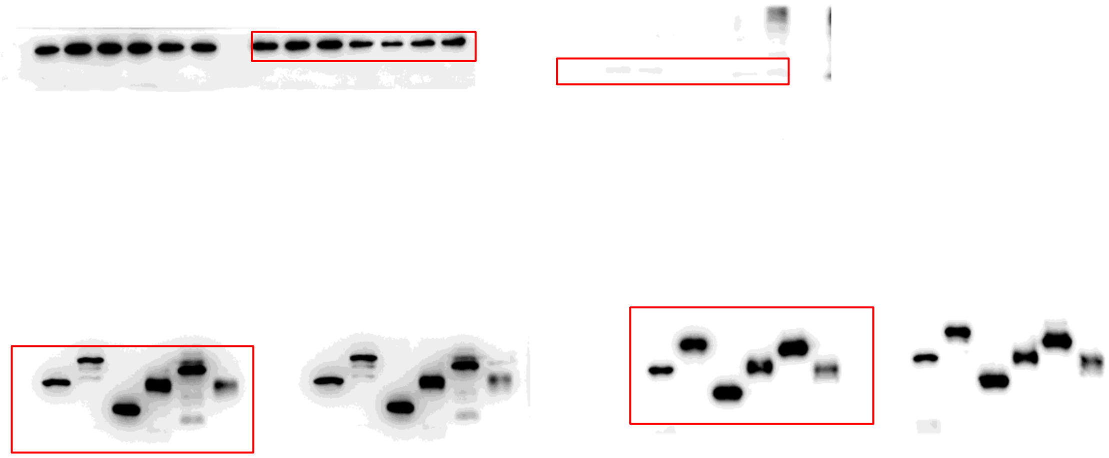

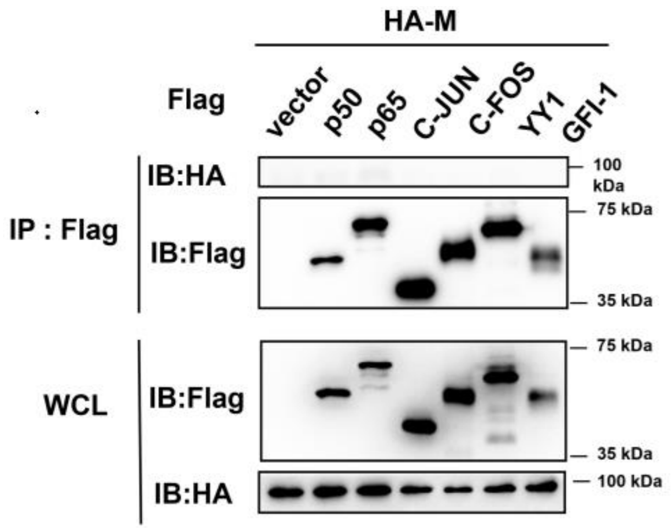

Figure E in Figure 2

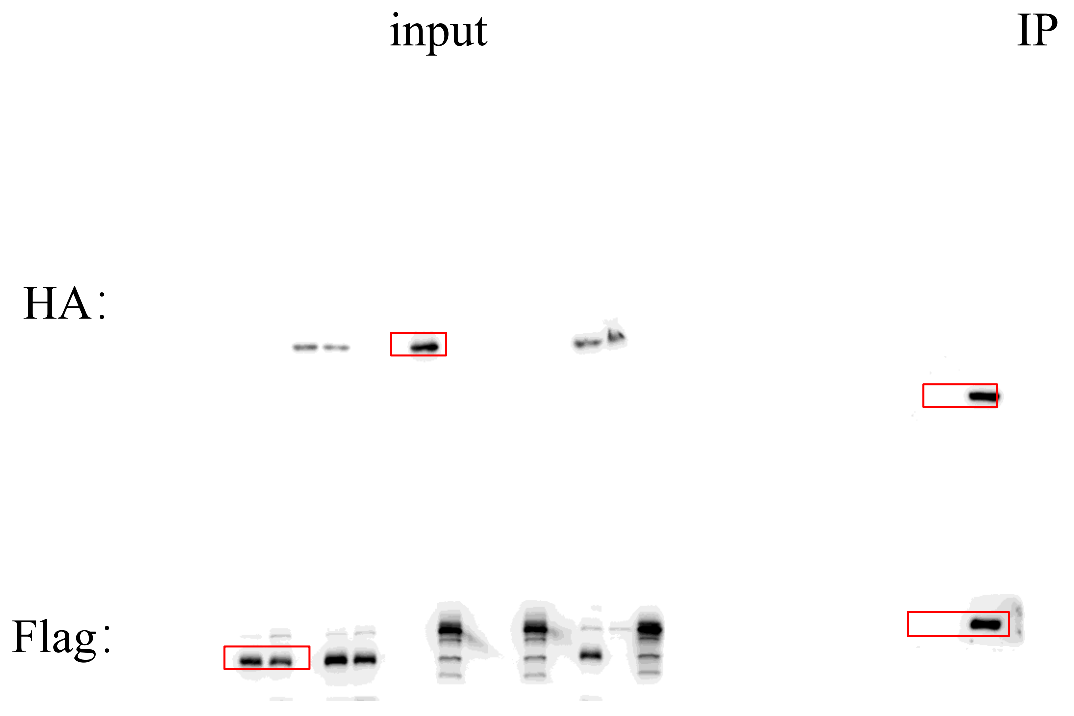

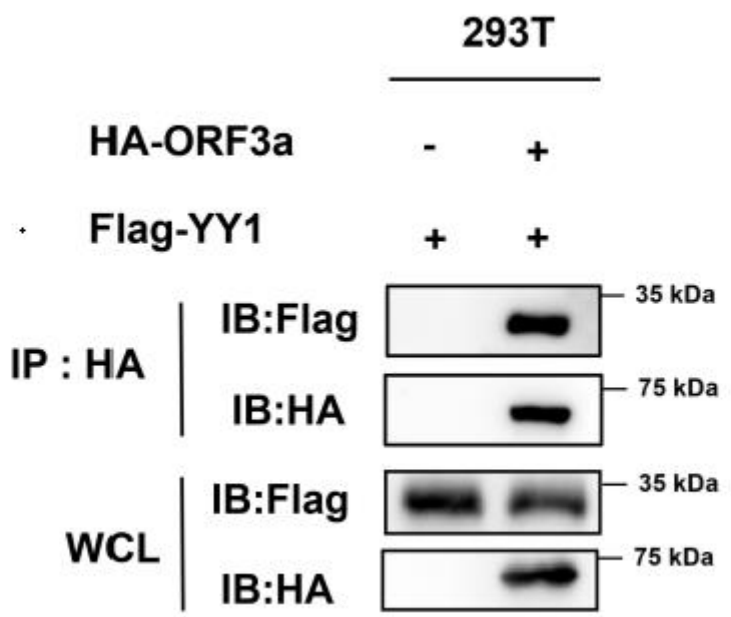

Figure F in Figure 2

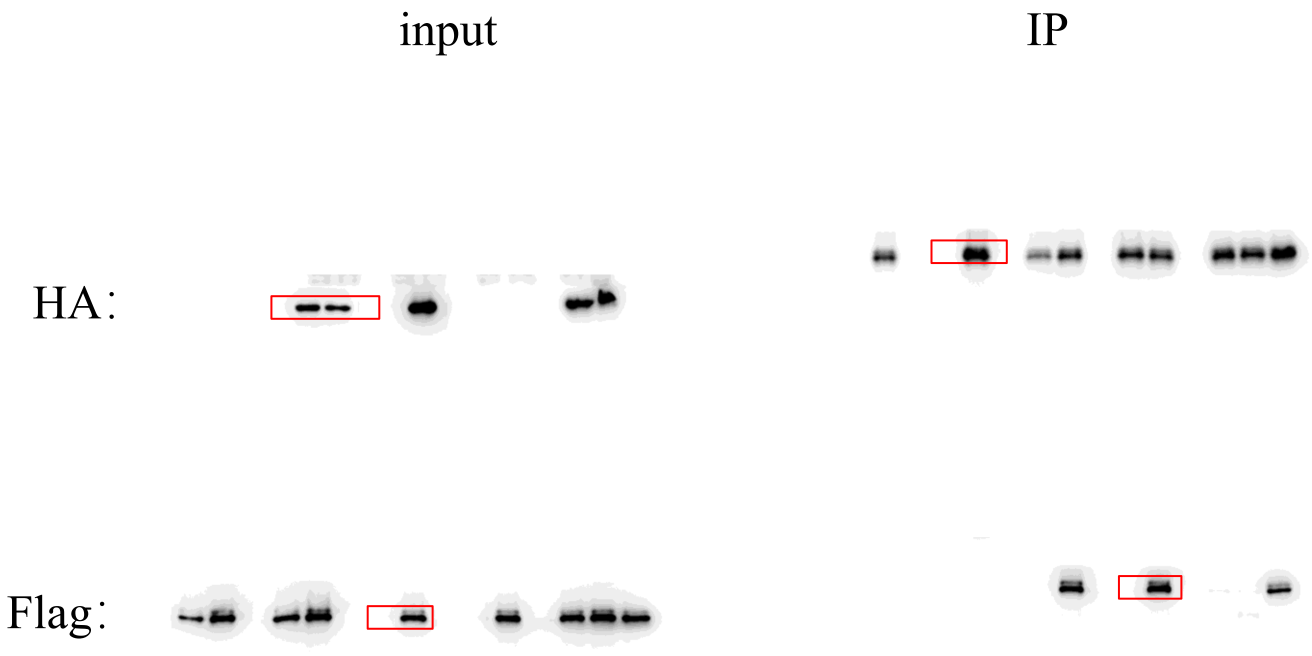

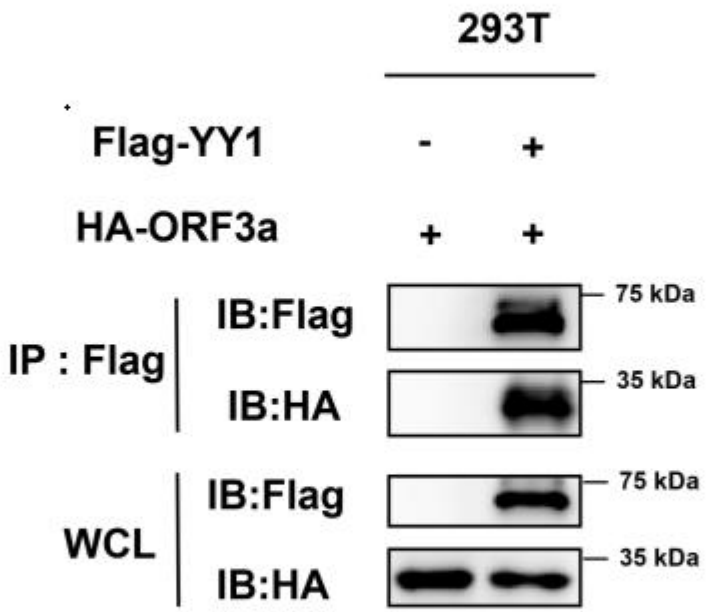

Figure H in Figure 2

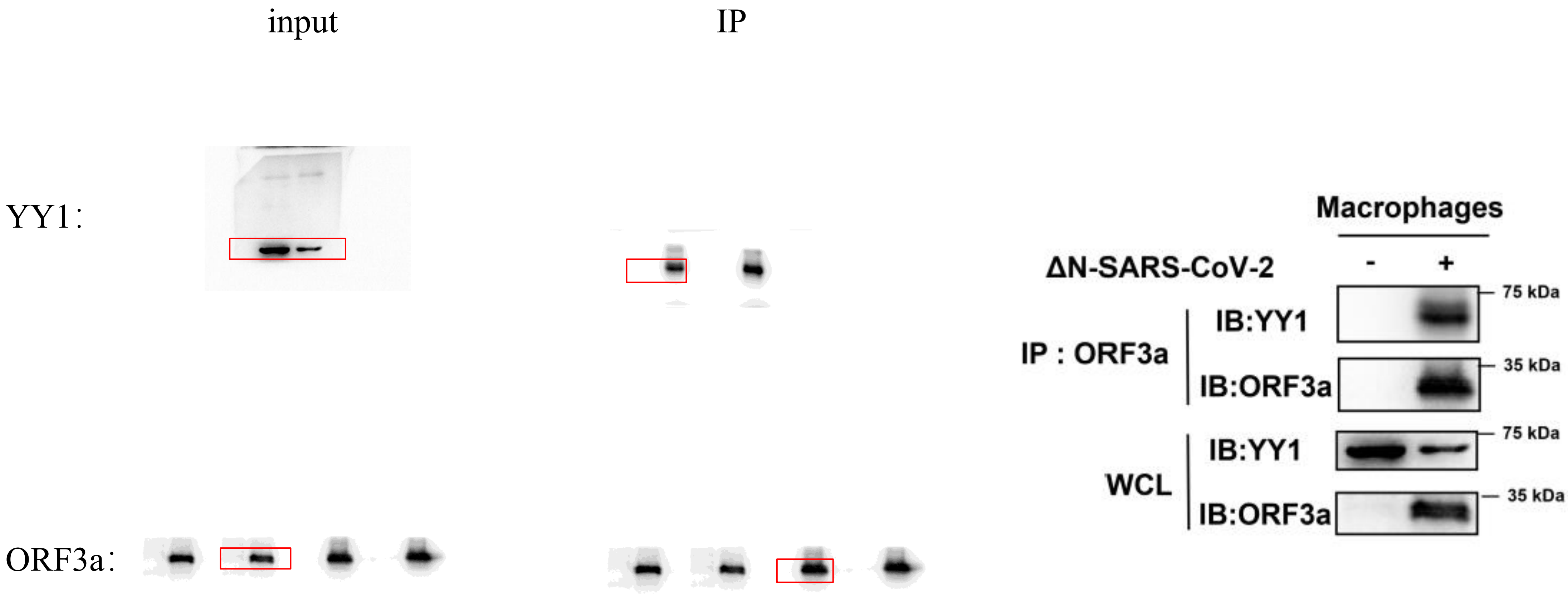

Figure I in Figure 2

ORF3a:

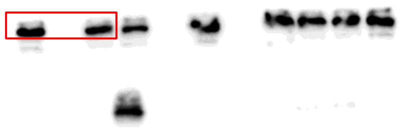

YY1:

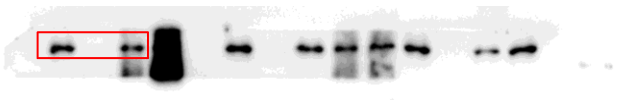

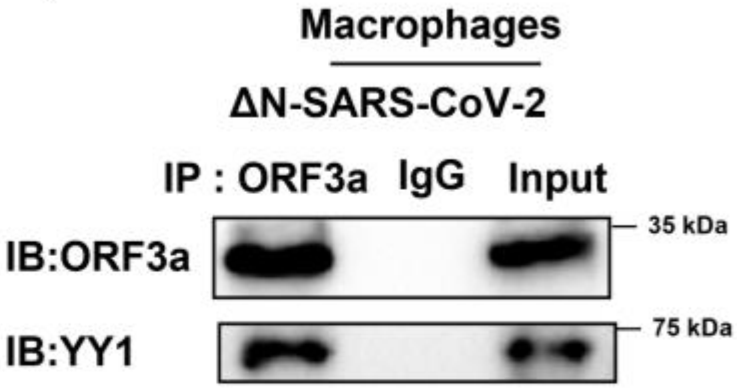

Figure K in Figure 2

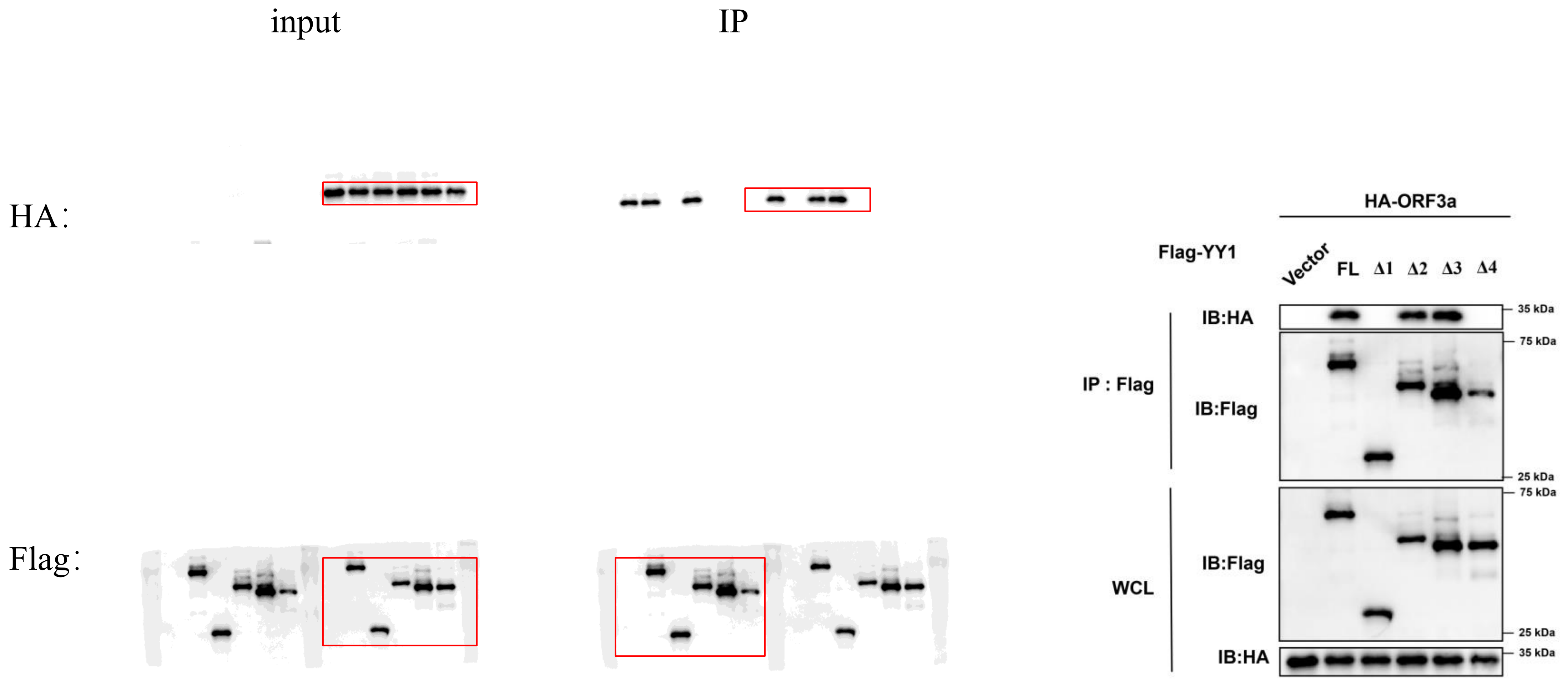

Figure A in Figure 3

Flag-YY1：

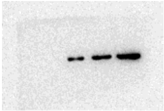

β-actin ：

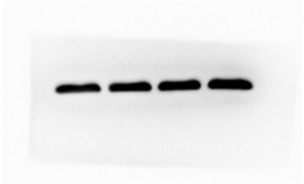

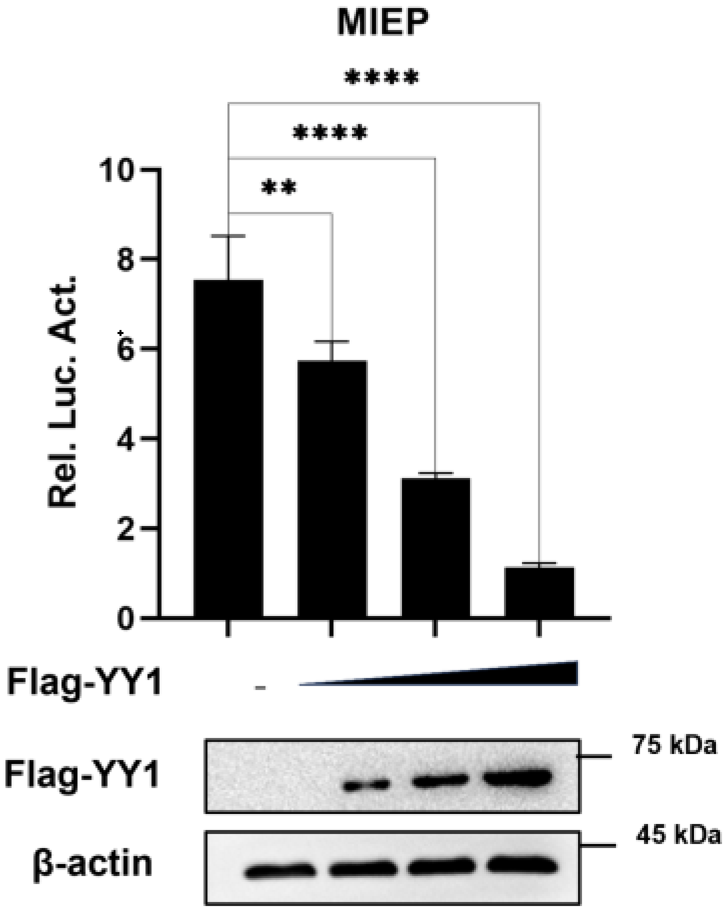

Figure B in Figure 3

Flag-YY1：

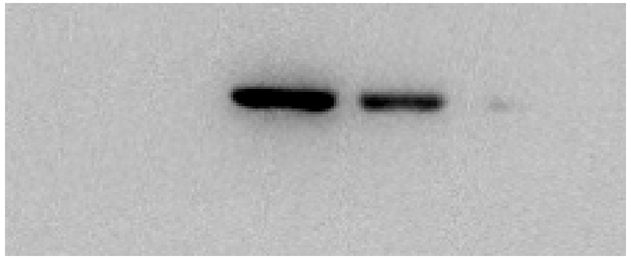

HA-ORF3a：

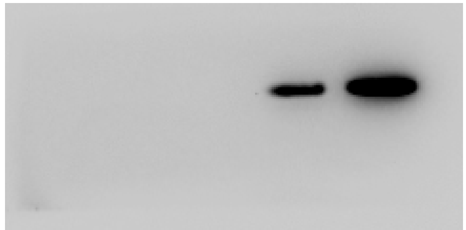

β-actin：

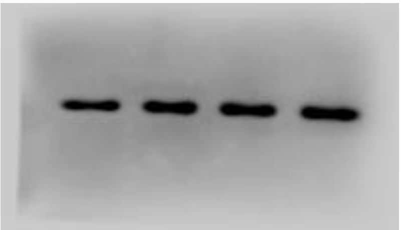

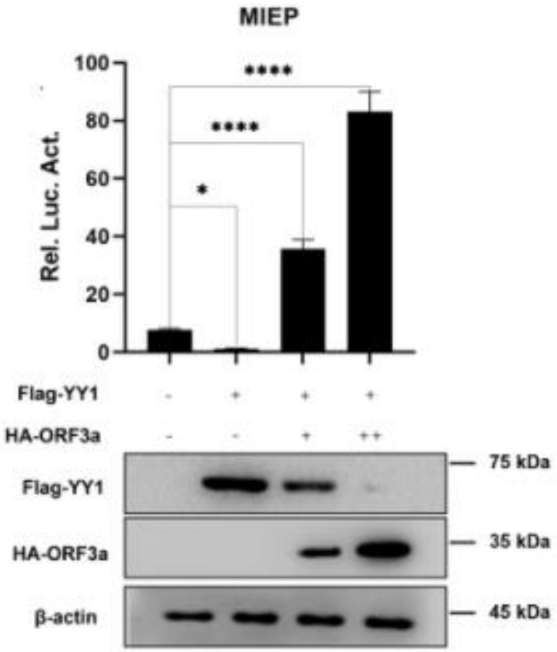

Figure C in Figure 3

HA-ORF3a:

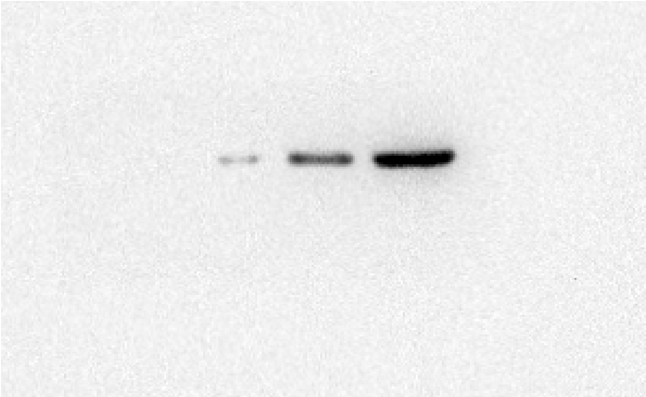

β-actin :

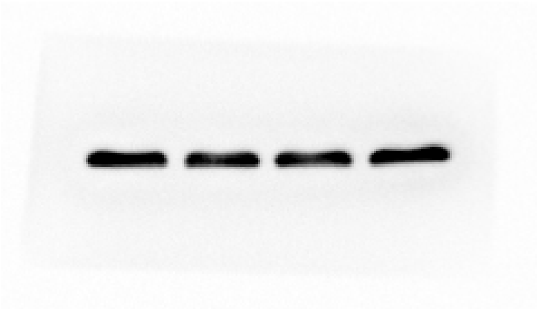

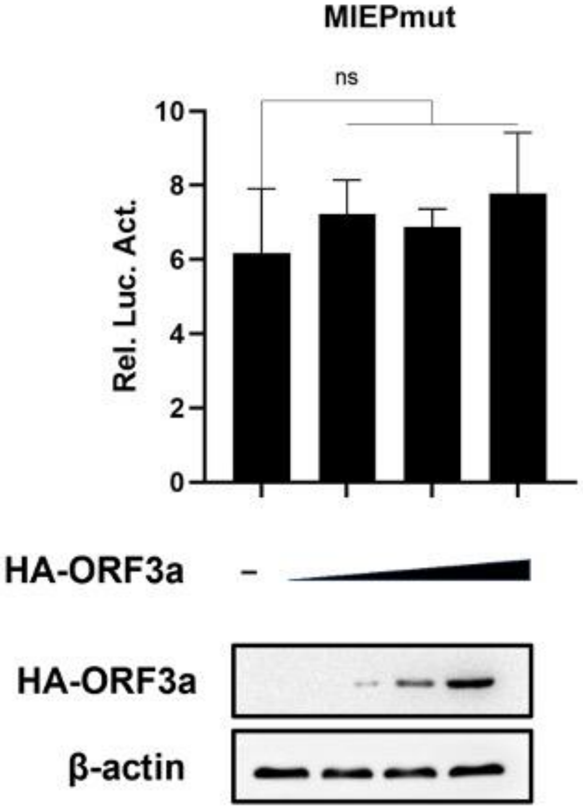

Figure D in Figure 3

HA-ORF3a：

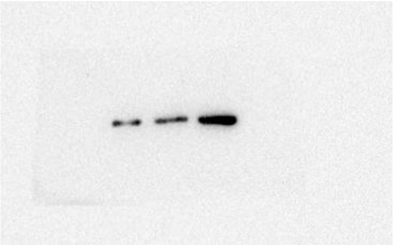

YY1：

β-actin：

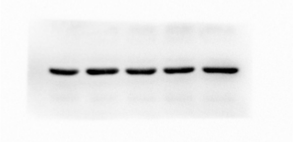

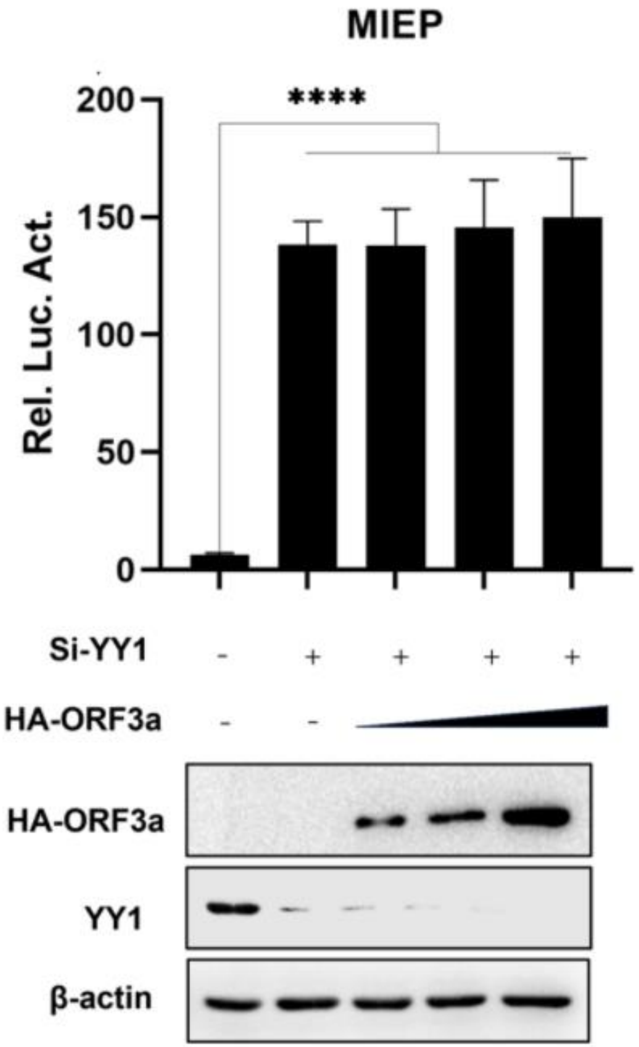

Figure E in Figure 3

Myc-Δ2：

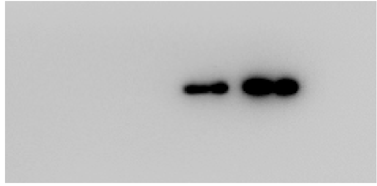

HA-ORF3a：

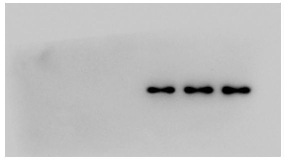

Flag-YY1：

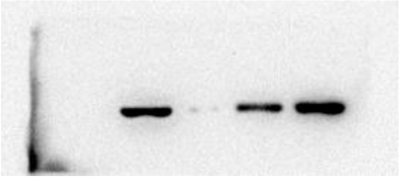

β-actin：

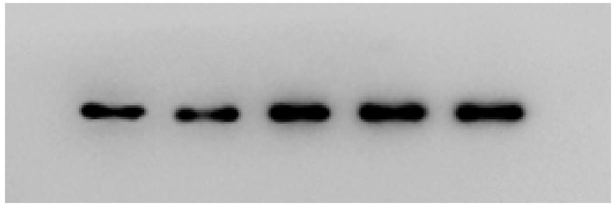

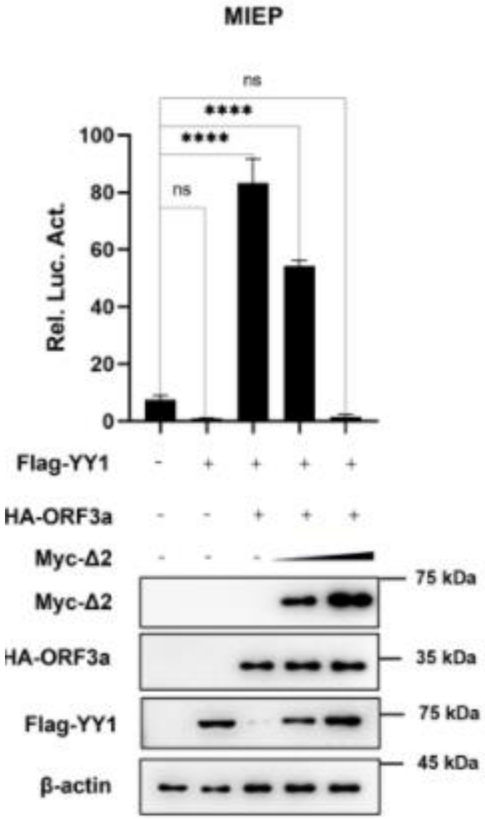

Figure A in Figure 4

Flag-YY1：

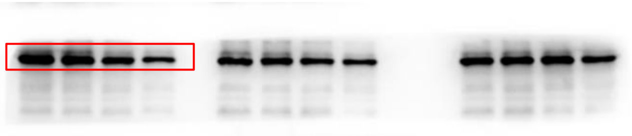

HA-ORF3a：

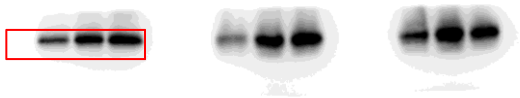

GAPDH：

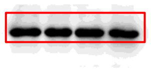

A

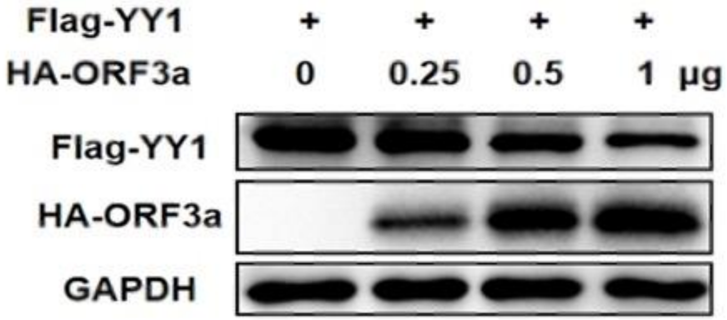

Figure B in Figure 4

YY1:

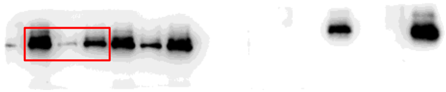

ORF3a:

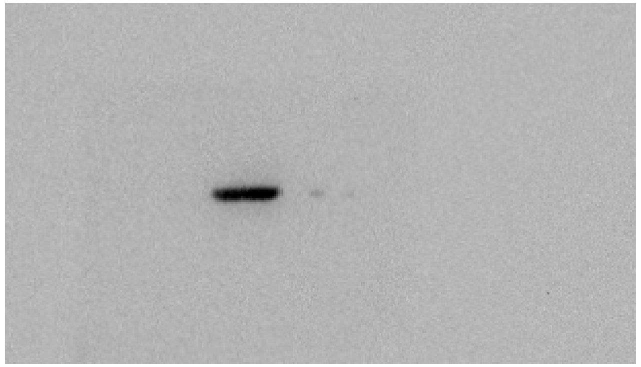

β-actin :

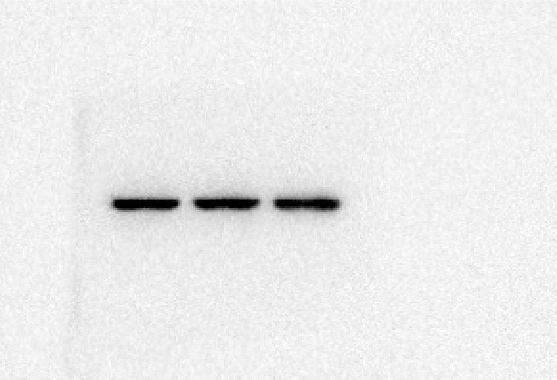

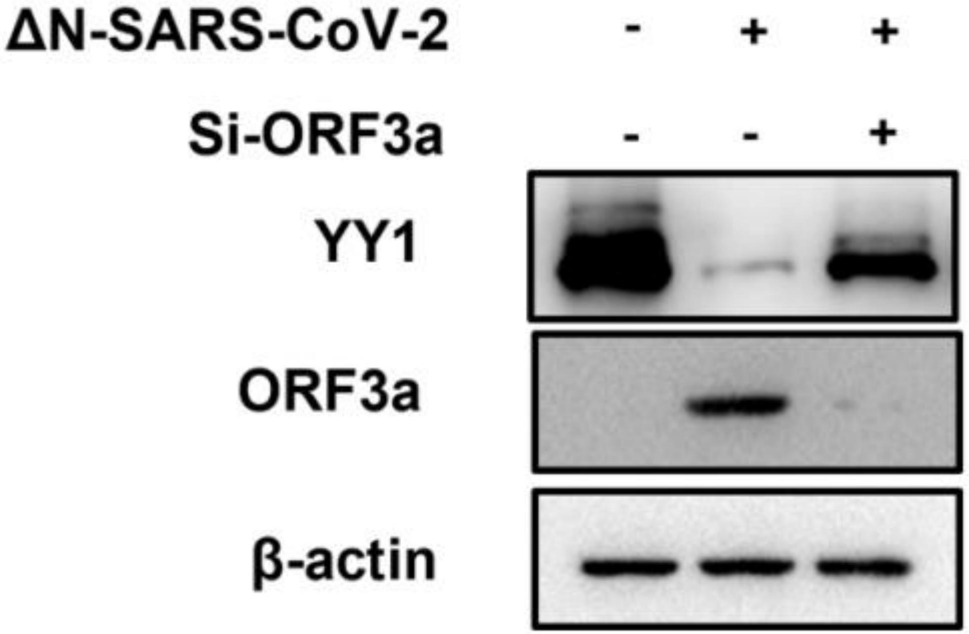

Figure C in Figure 4

input IP

HA:

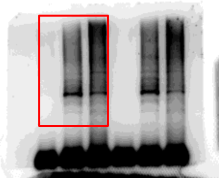

ORF3a:

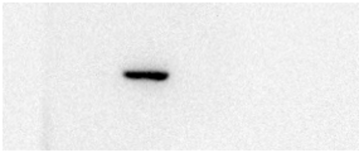

YY1:

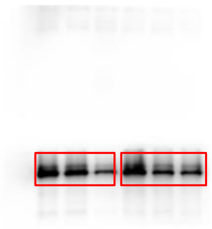

GAPDH:

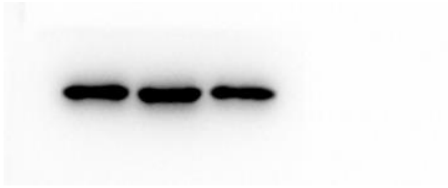

C

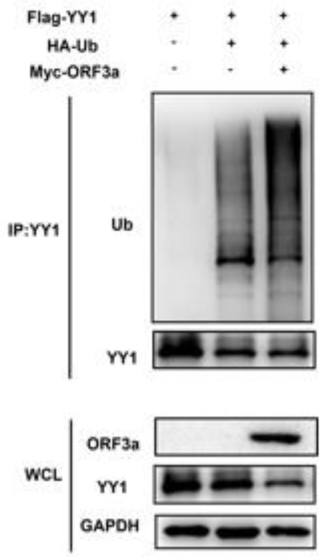

Figure D in Figure 4

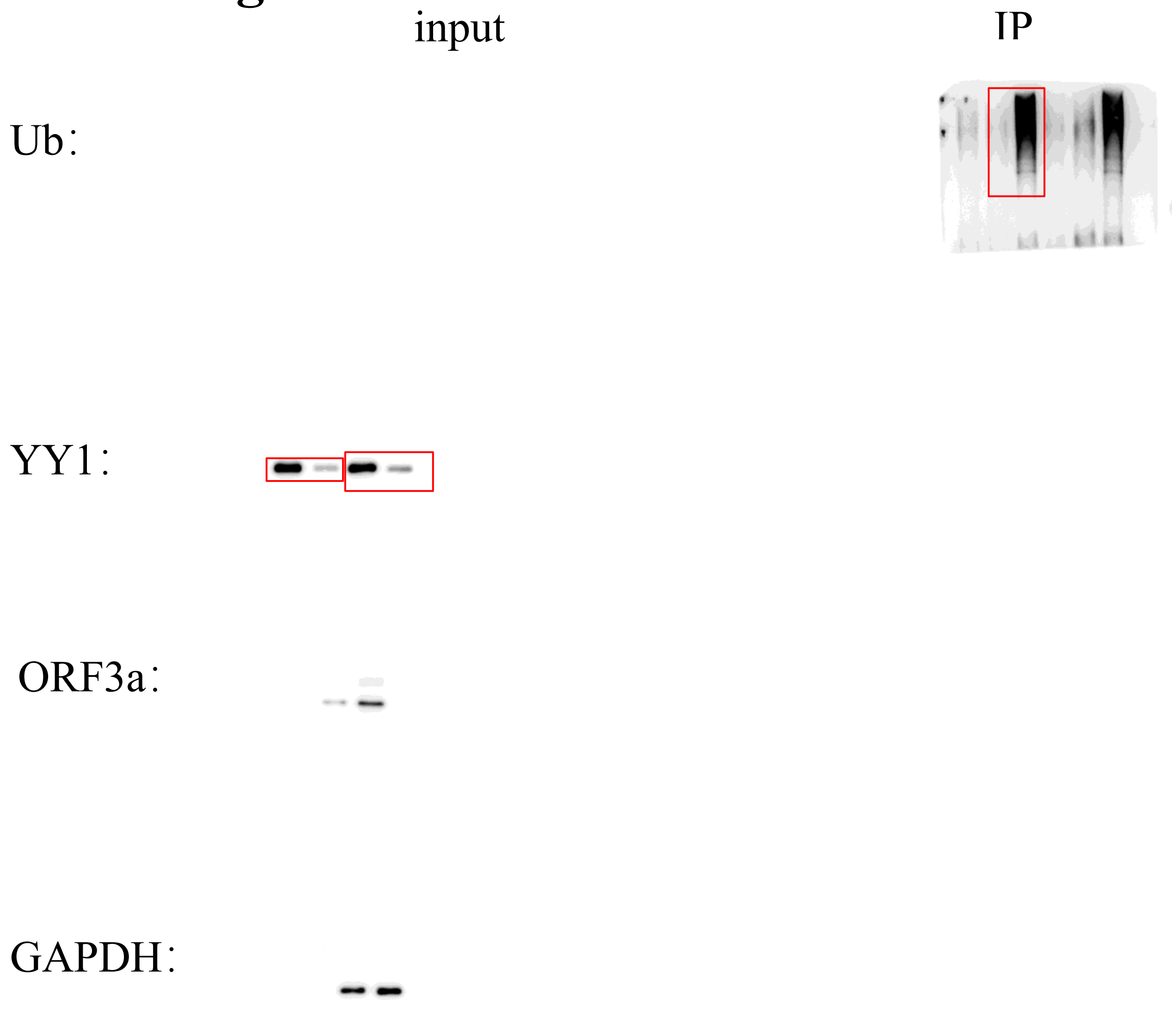

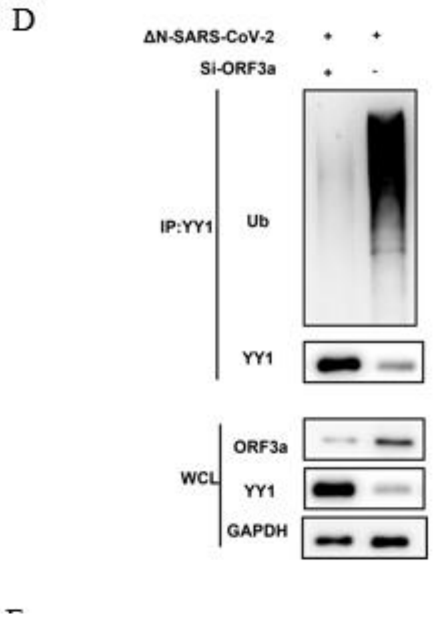

Figure E in Figure 4

YY1：

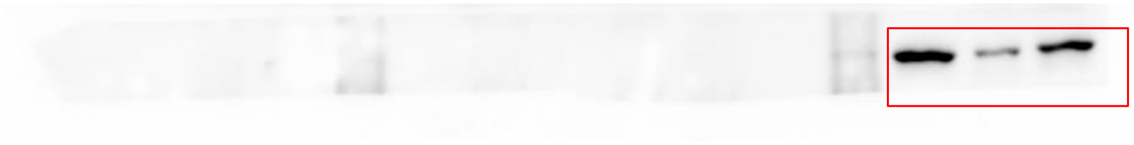

HA-ORF3a：

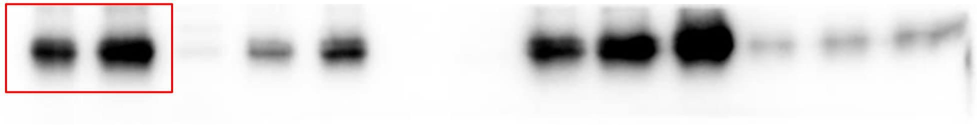

GAPDH：

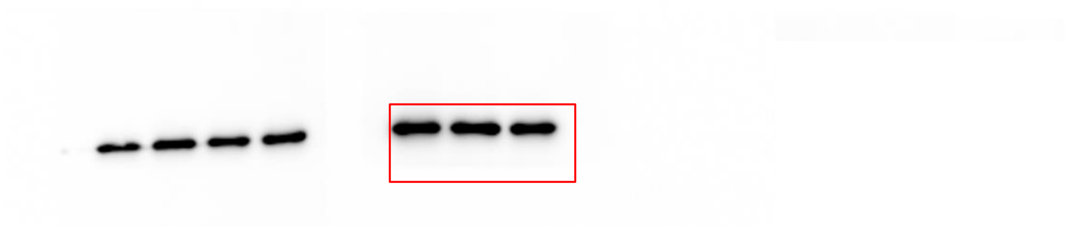

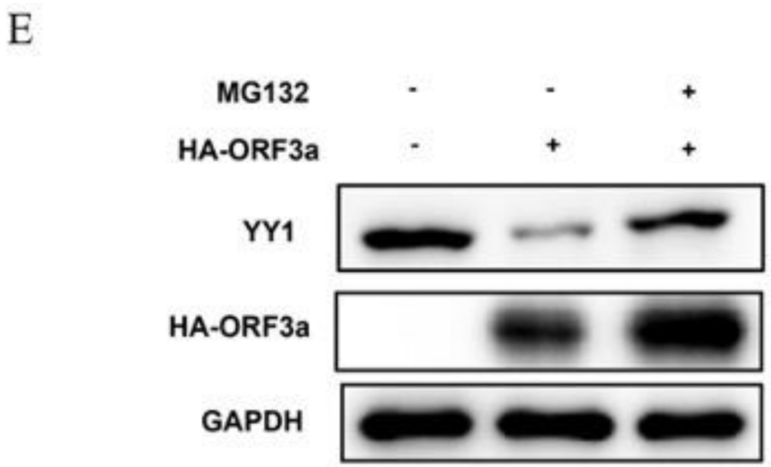

Figure F in Figure 4

YY1:

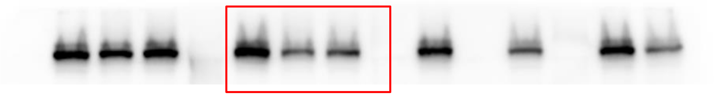

HA-ORF3a:

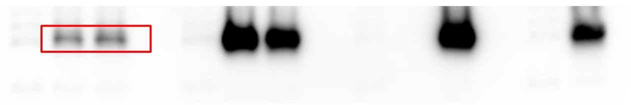

GAPDH:

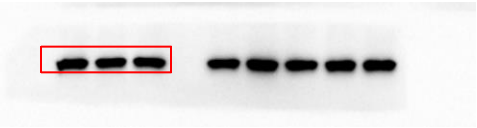

F

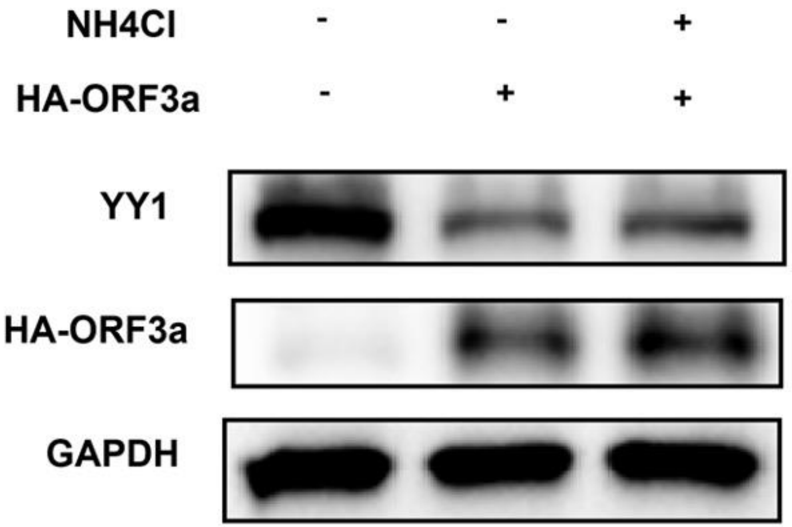

Figure G in Figure 4

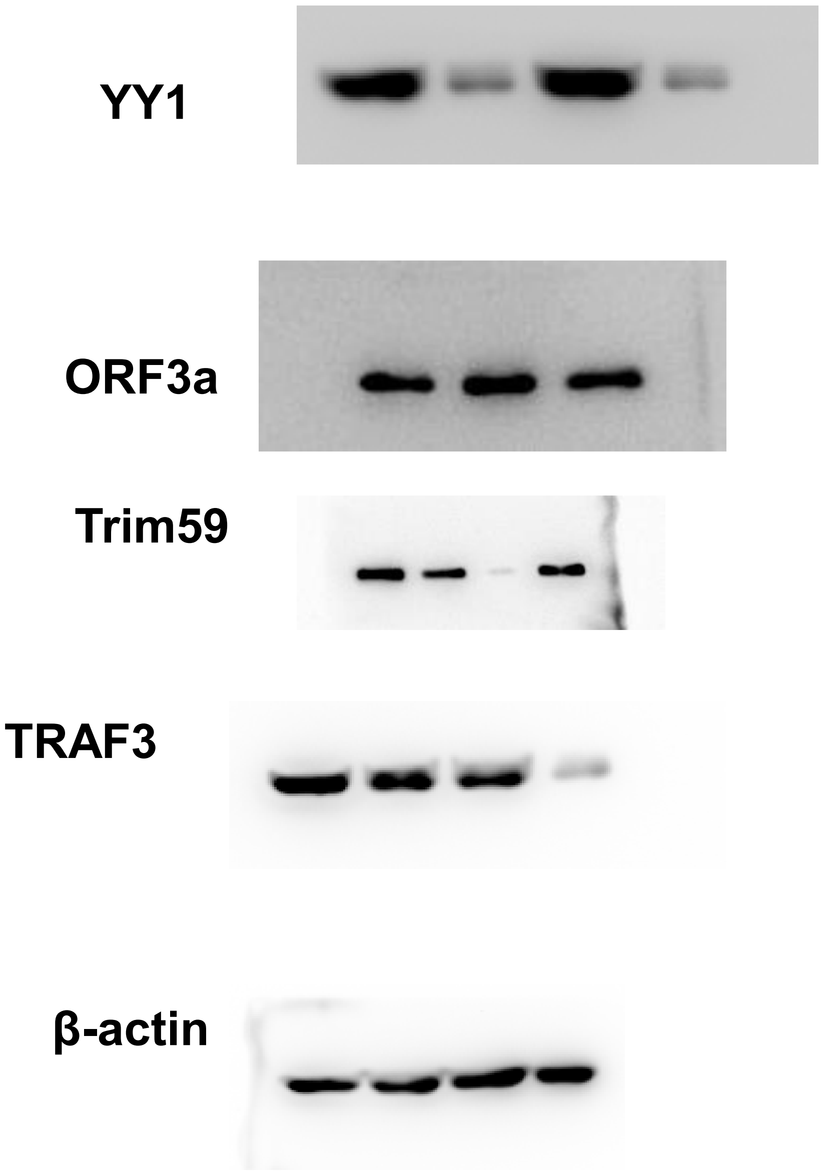

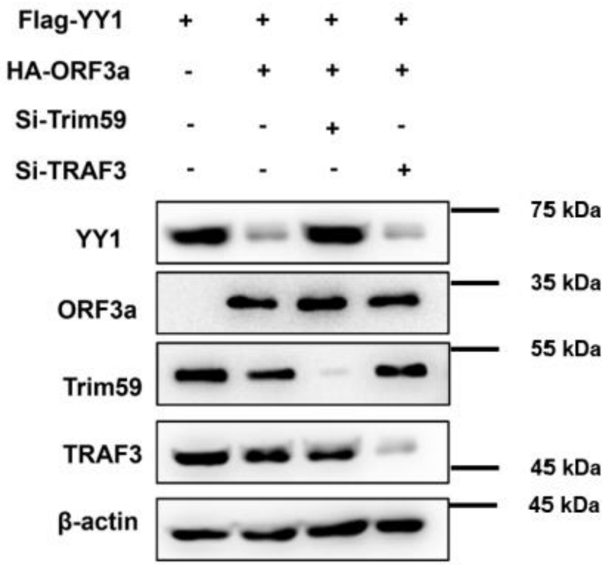

Figure H in Figure 4

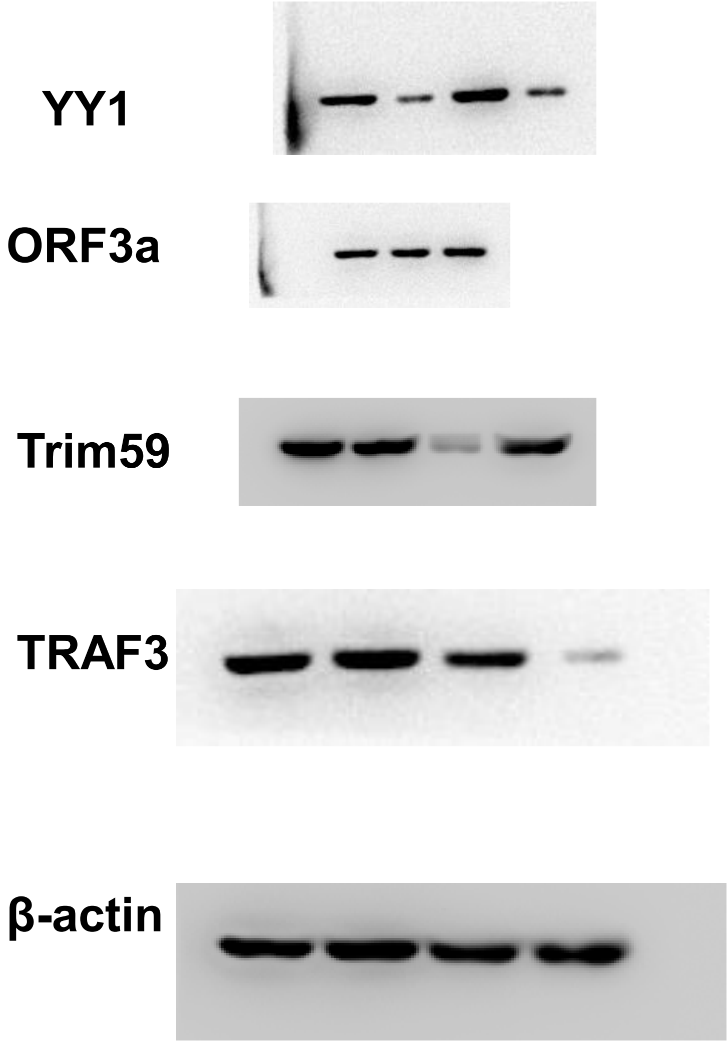

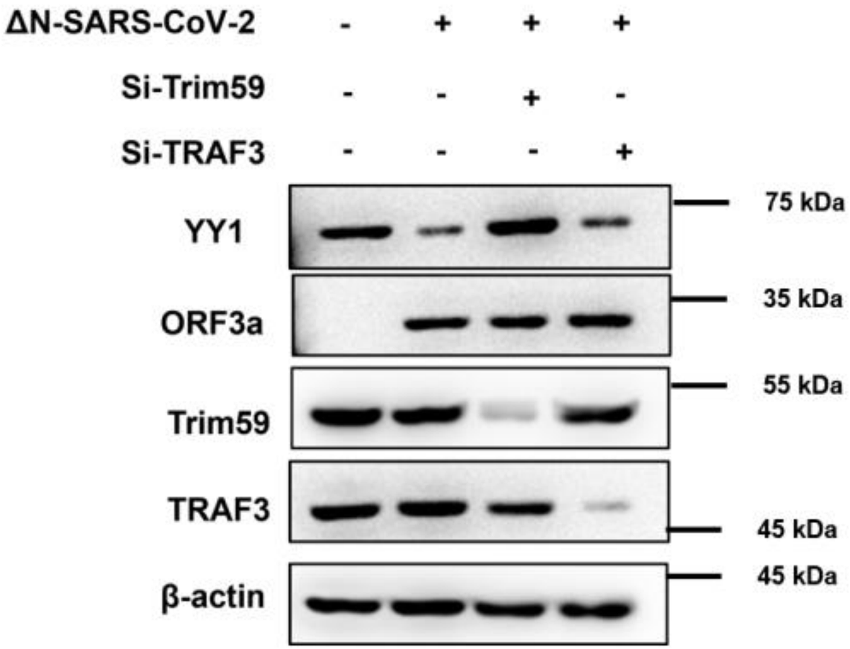

Figure E in Figure 5

IE1/2:

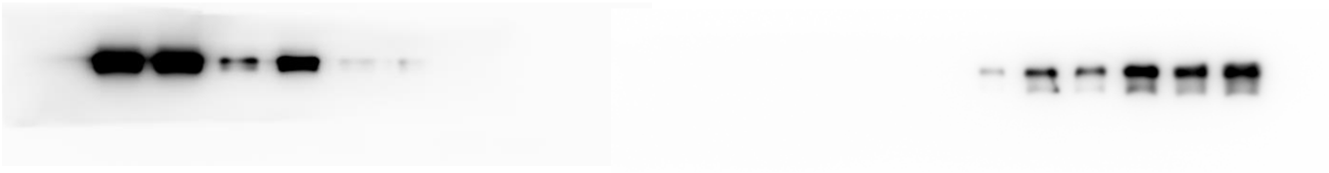

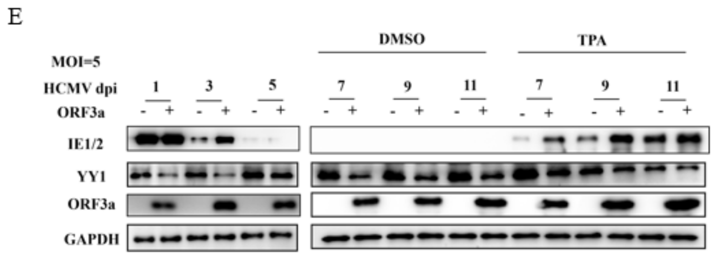

YY1:

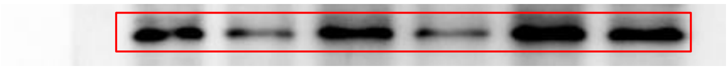

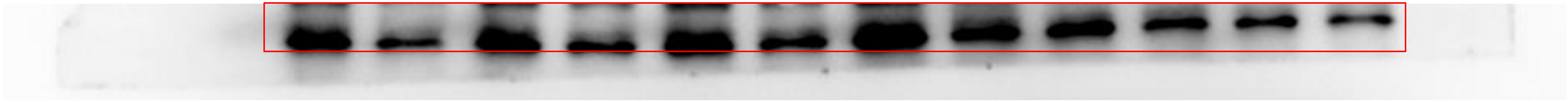

ORF3a:

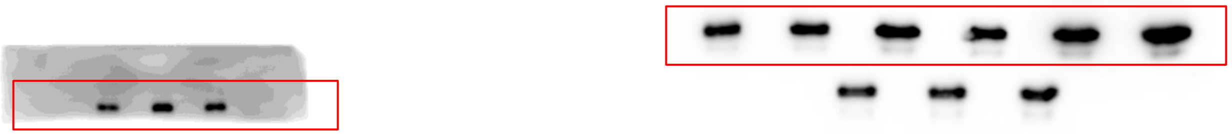

GAPDH:

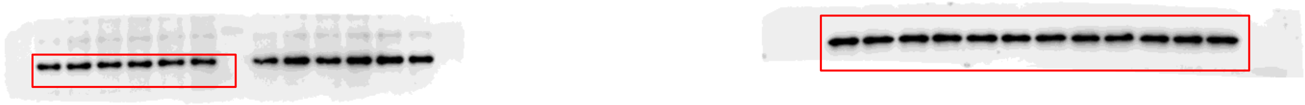

Figure G in Figure 5

IE1/2:

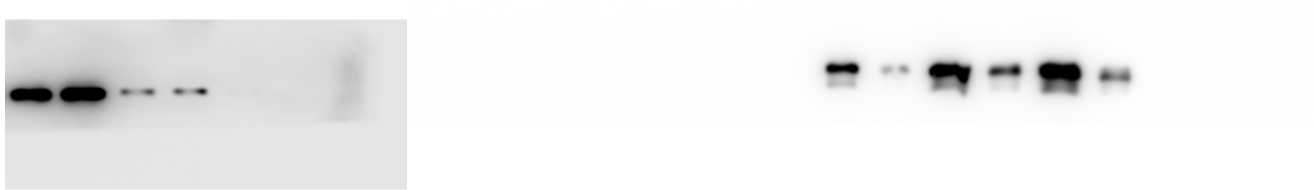

YY1:

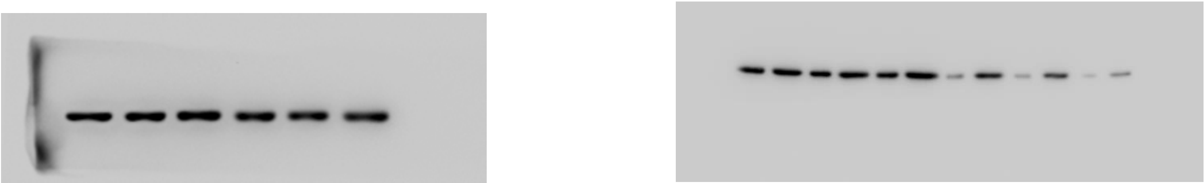

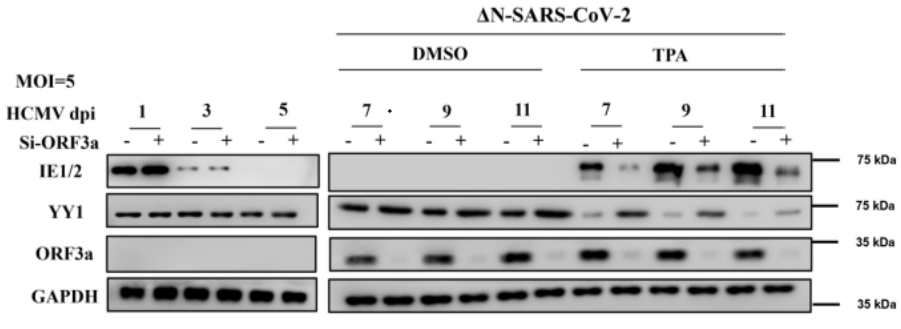

ORF3a:

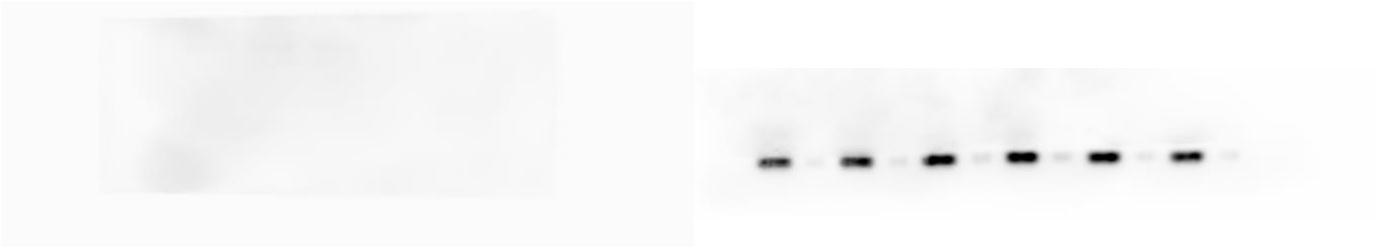

GAPDH

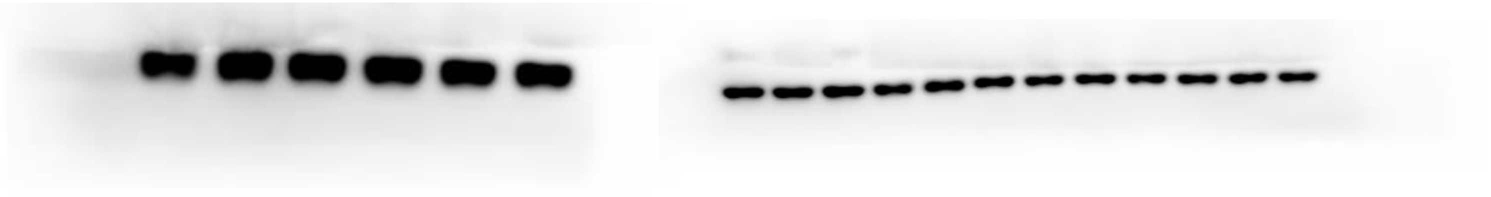

Figure I in Figure 5

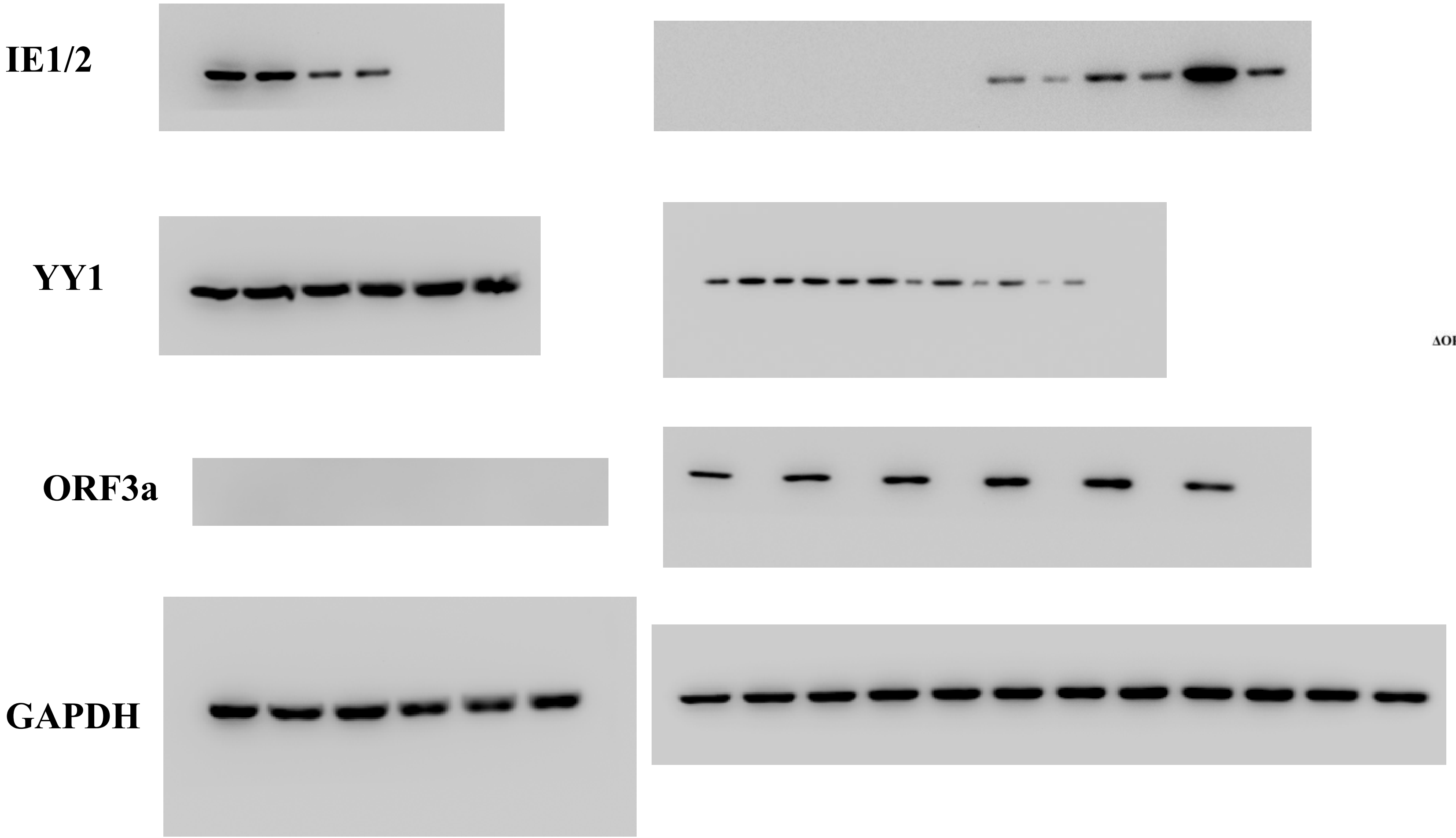

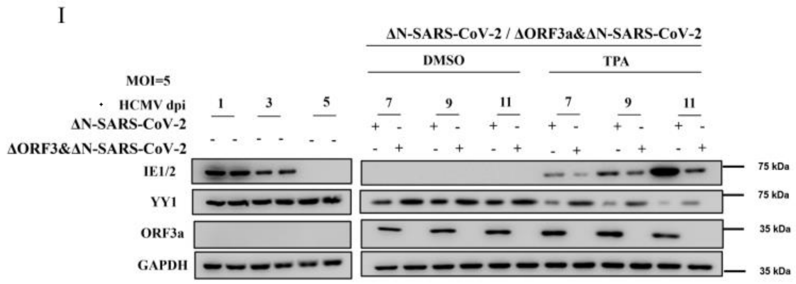

Figure S1

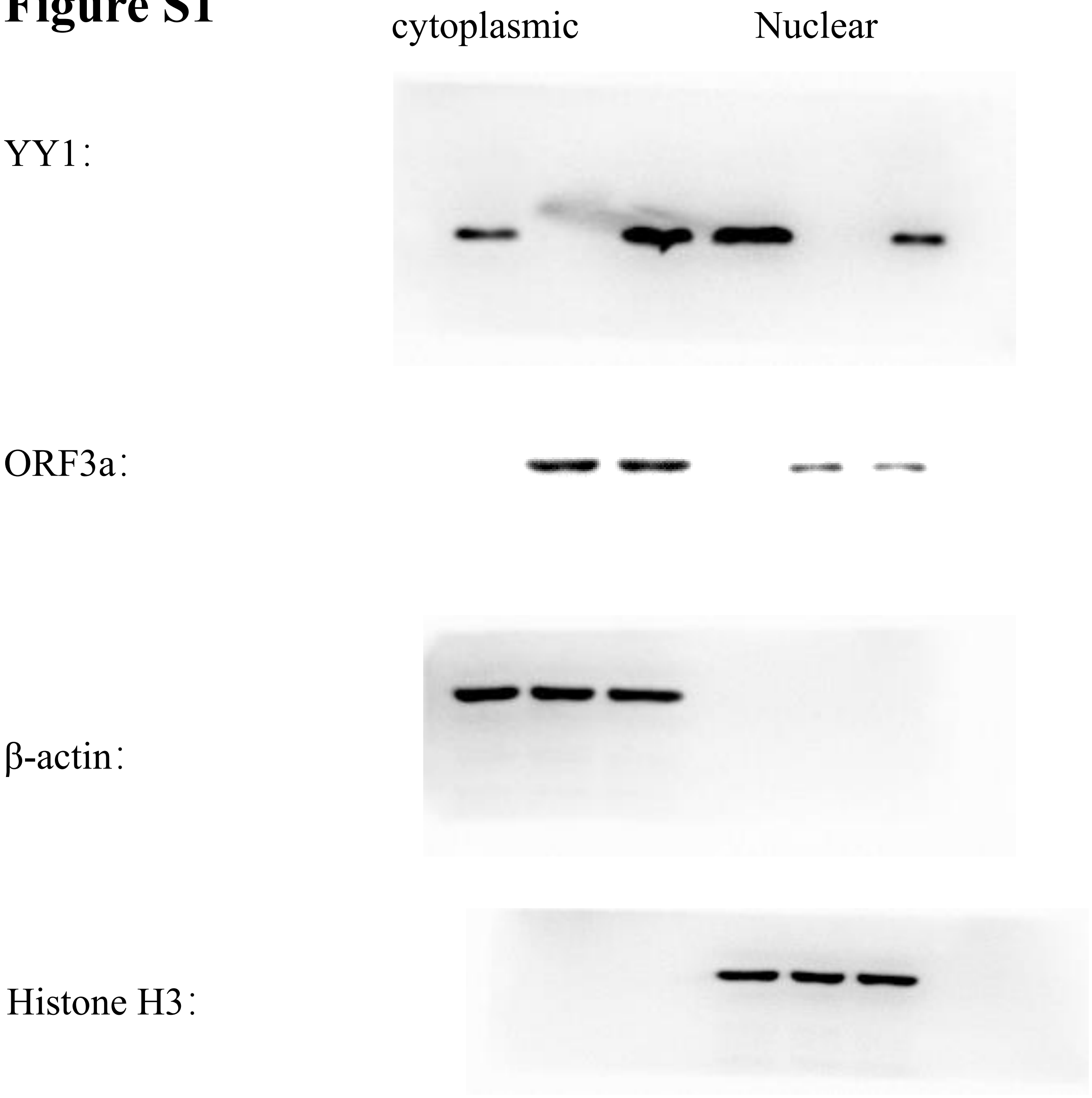
cytoplasmic
Nuclear
YY1:
ORF3a:
β-actin:
Histone H3:

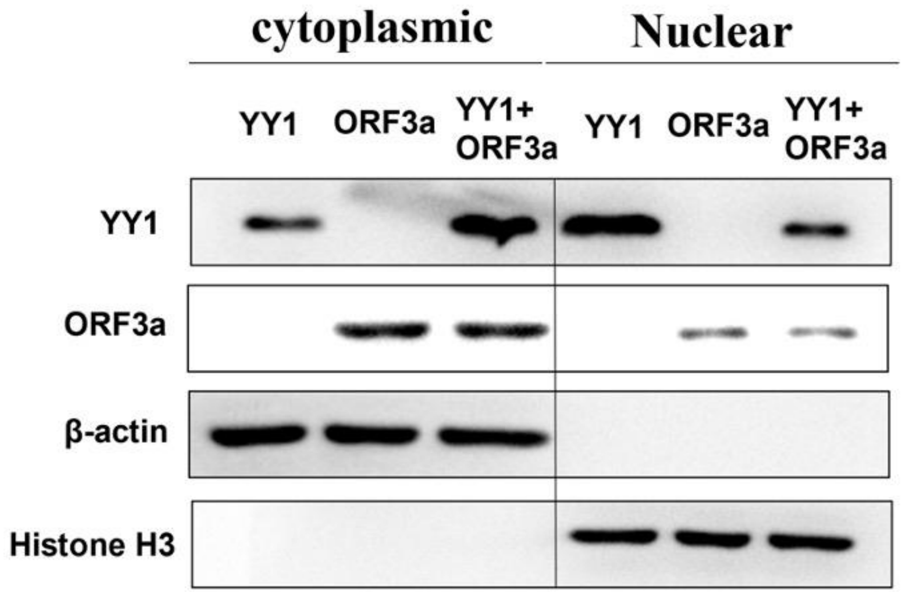
cytoplasmic
Nuclear
YY1
ORF3a
YY1+ ORF3a
YY1
ORF3a
YY1+ ORF3a
YY1
ORF3a
β-actin
Histone H3

Figure S2

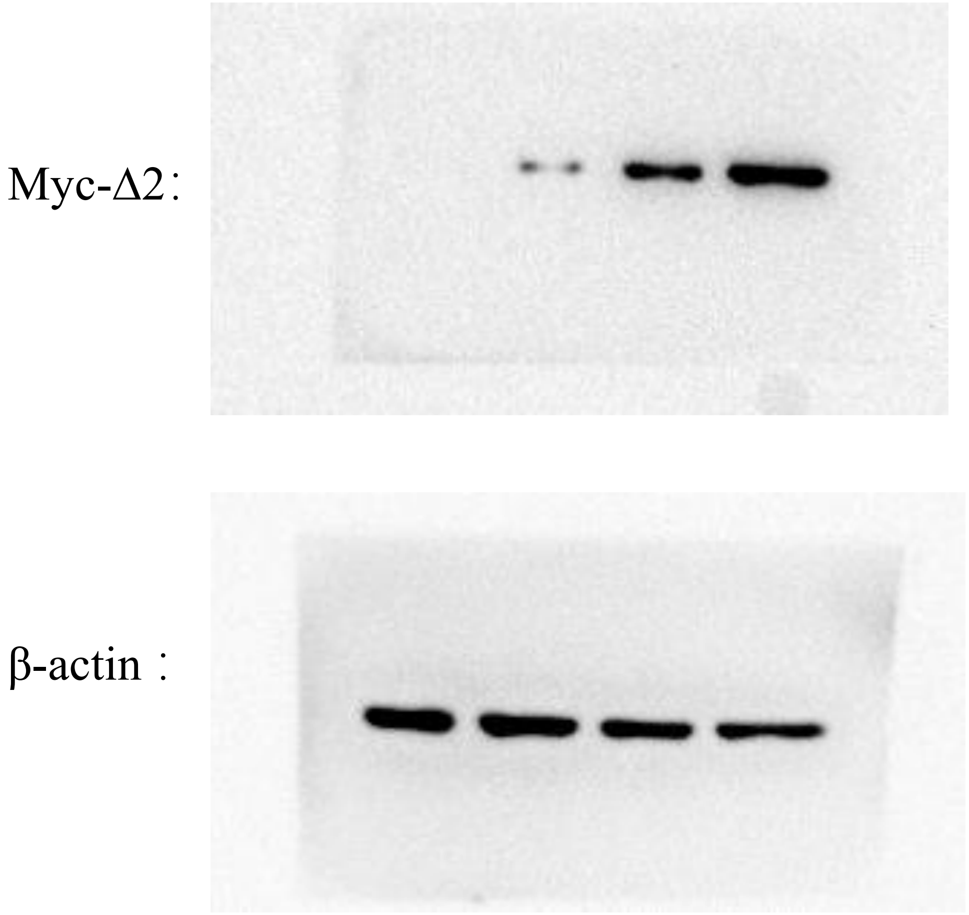

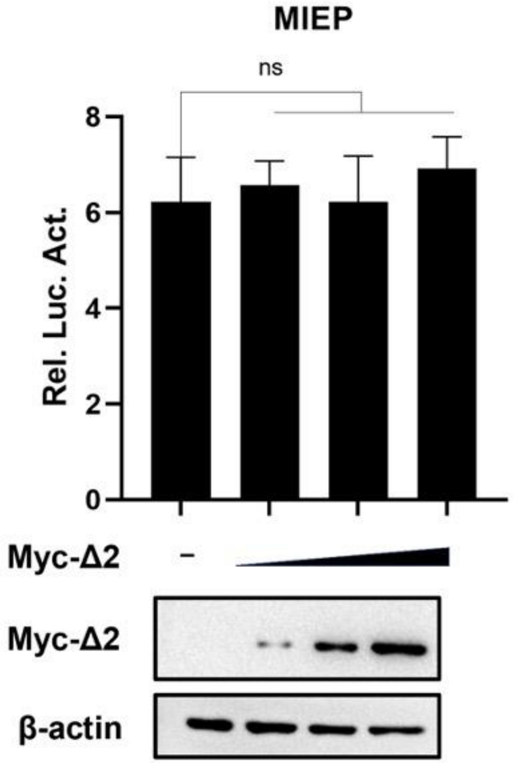

Figure S3

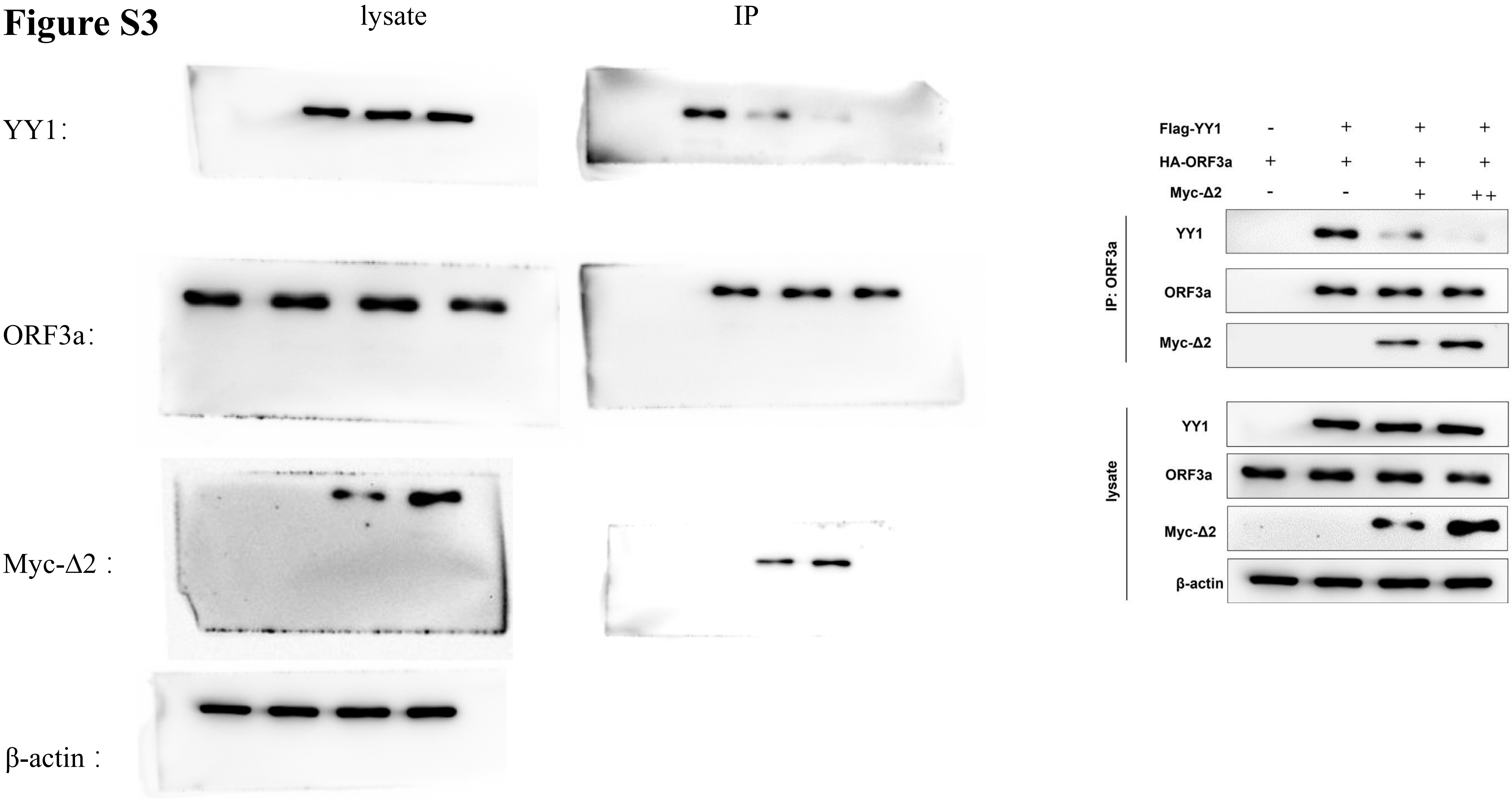
lysate
IP
YY1:
ORF3a:
Myc-Δ2 :
β-actin :
Flag-YY1 - + + +
HA-ORF3a + + + +
Myc-Δ2 - - + ++
IP: ORF3a
YY1
ORF3a
Myc-Δ2
lysate
YY1
ORF3a
Myc-Δ2
β-actin
